# Antiviral immune response reveals host-specific virus infections in natural ant populations

**DOI:** 10.3389/fmicb.2023.1119002

**Published:** 2023-03-16

**Authors:** Lumi Viljakainen, Matthias A. Fürst, Anna V. Grasse, Jaana Jurvansuu, Jinook Oh, Lassi Tolonen, Thomas Eder, Thomas Rattei, Sylvia Cremer

**Affiliations:** ^1^Ecology and Genetics Research Unit, University of Oulu, Oulu, Finland; ^2^Institute of Science and Technology Austria (ISTA), Klosterneuburg, Austria; ^3^Centre for Microbiology and Environmental Systems Science, Division of Computational System Biology, University of Vienna, Vienna, Austria; ^4^Institute for Medical Biochemistry, University of Veterinary Medicine Vienna, Vienna, Austria

**Keywords:** host-pathogen interactions, social insects, RNA viruses, RNAi response, siRNA, sRNA-seq

## Abstract

Hosts can carry many viruses in their bodies, but not all of them cause disease. We studied ants as a social host to determine both their overall viral repertoire and the subset of actively infecting viruses across natural populations of three subfamilies: the Argentine ant (*Linepithema humile*, Dolichoderinae), the invasive garden ant (*Lasius neglectus*, Formicinae) and the red ant (*Myrmica rubra*, Myrmicinae). We used a dual sequencing strategy to reconstruct complete virus genomes by RNA-seq and to simultaneously determine the small interfering RNAs (siRNAs) by small RNA sequencing (sRNA-seq), which constitute the host antiviral RNAi immune response. This approach led to the discovery of 41 novel viruses in ants and revealed a host ant-specific RNAi response (21 vs. 22 nt siRNAs) in the different ant species. The efficiency of the RNAi response (sRNA/RNA read count ratio) depended on the virus and the respective ant species, but not its population. Overall, we found the highest virus abundance and diversity per population in *Li. humile*, followed by *La. neglectus* and *M. rubra*. Argentine ants also shared a high proportion of viruses between populations, whilst overlap was nearly absent in *M. rubra*. Only one of the 59 viruses was found to infect two of the ant species as hosts, revealing high host-specificity in active infections. In contrast, six viruses actively infected one ant species, but were found as contaminants only in the others. Disentangling spillover of disease-causing infection from non-infecting contamination across species is providing relevant information for disease ecology and ecosystem management.

## 1. Introduction

Viruses and other pathogens are constantly exchanged between host individuals, be it by social interactions within species, predator–prey relationships between species, or by simply sharing a common environment (Boomsma et al., 2005; Fürst et al., 2014; French and Holmes, 2020). Thus, the presence of pathogens inside or on a host’s body is not sufficient to determine whether it is a disease-causing agent of this host species, or a mechanical infection, whereby the organism is a carrier of a pathogen without being infected itself. In addition to describing the variety of pathogens in a host, it is hence crucial to distinguish between active infections characterized by pathogen replication and a host immune response versus non-disease-causing contaminations, to determine the relevance of each pathogen for the disease dynamics and epidemiology within and across host populations.

The wide use of high-throughput RNA sequencing has recently started to provide unprecedented details on the variety of pathogens found in a host species. This includes so far less explored host-virus systems, exemplified by an extensive study of invertebrate viromes describing nearly 1,500 new RNA viruses (Shi et al., 2016) by sequencing of viral RNA fragments (approx. 150 base pairs [bp] length) and assembly to the complete virus genomes. Whilst providing an excellent overview over the viruses found in a host, this does not, however, allow to distinguish whether these are real infections or only contaminants. To allow for this distinction, Webster and colleagues (Webster et al., 2015) have recently combined such long-read sequencing with size-selected short-read RNA sequencing to also recover the 21–22 nucleotides long small interfering RNAs (siRNAs) of viral sequence (Bernstein et al., 2001), which are produced as host antiviral RNA interference (RNAi) response (Kemp and Imler, 2009).

In the RNAi response, the host immune system detects viruses by double-stranded RNA (dsRNA), which is either the infective viral stage (in double-stranded RNA viruses), a replication intermediate (in single-stranded RNA viruses) or sometimes triggered by an invading DNA virus (Son et al., 2015; de Faria et al., 2022). The dsRNA is bound by the host enzyme Dicer and cleaved to small RNAs typically of sizes 21–22 nt (Bernstein et al., 2001; Wilson and Doudna, 2013). The diced dsRNA is then loaded to the Argonaute protein generating an RNA-induced silencing complex (RISC), where one of the strands is discarded while the other can then bind in a sequence-specific manner to more virus, leading to perpetuating cleavage to disarm viral replication (Wilson and Doudna, 2013). This efficiently reduces the viral population in a host. The host immune response against an infective and actively replicating virus therefore produces virus-sequence-specific siRNAs in the host’s cells which can be used as markers for an active viral infection (Webster et al., 2015). When Webster and colleagues used this combination of fragment size and sequence information in the well-studied *Drosophila melanogaster* system – currently the main model for studying individual immune responses in invertebrates (Buchon et al., 2014) – they were able to detect more than 20 novel viruses which represented active infections (Webster et al., 2015). Whilst highly abundant viruses are generally expected to represent host infection rather than contamination, this method is particularly powerful to define the infectivity of viruses found at low abundance.

Another important defense pathway, the PIWI pathway, produces a population of small RNAs (piRNA) and is mainly active in the germline to protect against transposable elements, albeit in *Aedes* spp. mosquitoes, also viruses can trigger this pathway (Blair, 2019; Kolliopoulou et al., 2019). Although there is some overlap in the small RNA populations produced by both pathways, the majority of piRNAs (24 to 35 nt, Hirakata and Siomi, 2016) are slightly larger than the siRNAs (predominantly 21–22 nt, Gammon and Mello, 2015), and have a preference for uridine (U) at the 5′-base unlike the siRNAs, which helps in disentangling the small RNAs resulting from the different pathways by bioinformatic processes.

In this study, we aimed to determine active viral infections that represent a disease threat and cause the RNAi host response in social insects, particularly ants, which have a functional RNAi pathway (Lu et al., 2009). Like all social species, social insects face a high risk of transmission of infectious disease due to the close social contact between hosts (Schmid-Hempel, 1998; Cremer et al., 2007). Within the social insects (the social bees and wasps, the ants and the termites), viruses have been intensively studied in the honeybee and bumblebees, due to their ecological and economic importance as pollinators (for a review, see Grozinger and Flenniken, 2019). Bees are infected with a wide diversity of viruses. Transmission occurs either directly, mostly at flowers, which constitute shared food sources to many colonies and species – therefore often being ‘disease hotspots’– but also by worker drift to other colonies (Geffre et al., 2020), or *via* vectors, such as the *Varroa* mite (Boomsma et al., 2005; Chen et al., 2006). Bee viruses are hence transmitted within and among bee species (Fürst et al., 2014; Alger et al., 2019), as well as to other insects, including ants (Celle et al., 2008; Levitt et al., 2013; Gruber et al., 2017; Viljakainen et al., 2018; Schläppi et al., 2020). Viruses in bees may lie dormant or cause symptoms like deformed wings, paralysis or death, often causing important diseases such as the sacbrood disease in managed honeybee populations, but also the native bees and bumblebees, to which spillover regularly occurs in the field (Fürst et al., 2014; McMahon et al., 2015).

Ants and termites are less well studied, but we expect very different viral infection patterns than in bees due to their different ecology. They are highly territorial and feed exclusively within territories that they rigorously defend against neighboring colonies, making cross-colony transmission very rare. Ants and termites are therefore expected to be less affected by viruses and bacteria, whose infectious stages can often only survive for a very limited period outside of a host in the environment (except if being able to produce long-lasting stages like spores, see Boomsma et al., 2005). Instead, infection of ants and termites frequently occurs *via* long-lasting infectious stages, picked up from the environment, such as bacterial and fungal spores from sporulating cadavers or from the soil (Boomsma et al., 2005; Cremer et al., 2018). Yet, as many ant species are scavengers and collect dead insects to feed their larvae, they can also pick up infections from virus-infected prey or when in vicinity to virus-infected other insects, like beehives (Porter et al., 2016; Schläppi et al., 2020). Several studies on ants, most using molecular screening approaches, have reported that ants carry viruses (as found in 13 ant species, Supplementary Table 1; Baty et al., 2020), but determination which of them cause active infection is missing in most cases. Data on viral pathogenicity are so far only available for the well-studied invasive fire ant, *Solenopsis invicta*, where viruses provide a useful tool in biocontrol (Valles and Hashimoto, 2009; Manfredini et al., 2016; Oi et al., 2019; Valles et al., 2022).

The social lifestyle of social insects has strong effects on disease dynamics. Colony members are typically highly related and live in dense communities where they frequently interact with each other to share information and food. These factors offer ideal conditions for pathogen transmission and persistence within a colony, but they also allow for coordinated and highly sophisticated cooperative disease defenses, providing “social immunity” to the colony (Cremer et al., 2007, 2018). Disease outbreaks are rare, due to efficient nest sanitation by antimicrobial compounds (Christe et al., 2003; Pull et al., 2018a), active removal of disease vectors like the *Varroa* mite (Evans and Spivak, 2010), grooming of contaminated nestmates (Rosengaus et al., 1998; Hughes et al., 2002; Theis et al., 2015), removal and/or disinfection of infected brood (Rothenbuhler, 1964; Tragust et al., 2013; Pull et al., 2018b) and changes in the social interaction network reducing its disease transmission properties after infection (Stroeymeyt et al., 2018). Social immunity is complemented by individual immunity of the insects, particularly their physiological immune system (Siva-Jothy et al., 2005; Konrad et al., 2012; Buchon et al., 2014), yet details on the expression and consistency of the RNAi response in ants is still missing, as is a comprehensive overview over their natural infection patterns.

To gain better insight into the viral infection patterns across ant species in the field, we studied one representative species each of the three major subfamilies of ants, the Argentine ant *Linepithema humile* (Dolichoderinae), the invasive garden ant *Lasius neglectus* (Formicinae) and the common red ant *Myrmica rubra* (Myrmicinae). For each ant species, we investigated RNA virus diversity, abundance and infectivity in three different populations to determine if infection patterns are species-specific or whether they follow regional patterns of infection. This choice of ants also allowed us to evaluate a possible effect of social structure on virus infections. All three studied ants are invasive species, yet we sampled only *Li. humile* [native to South America (Erickson, 1971)] and *La. neglectus* [putative origin from the Black Sea area (Seifert, 2000)] from their introduced range in Southern Europe. Here, we also collected *M. rubra*. This species is native in these Eurasian populations from where it has been introduced to North America and Canada (Groden et al., 2005). Whilst forming small colonies with high territorial aggression in their native ranges, invasive ants form huge supercolonies in their introduced range (Suarez et al., 2001; Giraud et al., 2002; Ugelvig et al., 2008). Supercolonies are networks of nests, where the lack of aggression between individuals from the same supercolony enables constant exchange between nests and growth to enormous sizes (Giraud et al., 2002; Cremer et al., 2008). We expect these interactive networks to likely facilitate viral transmission across nest borders (Ugelvig and Cremer, 2012; Cremer, 2019). We applied the dual sequencing approach of long (~150 bp) and short (~30 bp) RNA sequencing on these ants, to determine their viral repertoire and overlap of active viral infections versus passive contaminants only, which has important implications for viral disease dynamics within and between natural populations of ants.

## 2. Materials and methods

### 2.1. Ant collection

**Figure 1:**
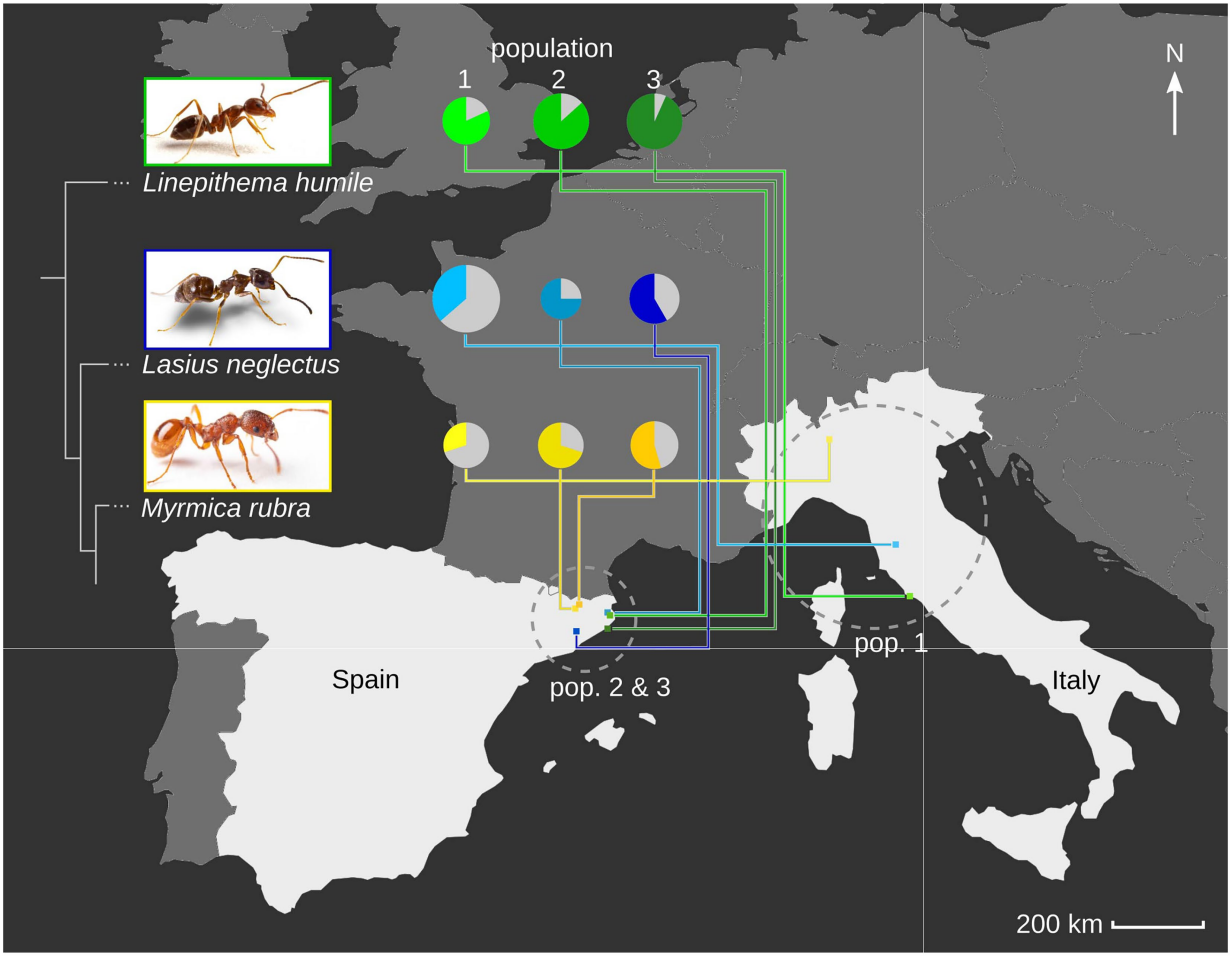
Ant host species and population viral diversity. We determined the viral repertoire of three populations each of three ant species, *Linepithema humile* (Dolicoderinae, green), *Lasius neglectus* (Formicinae, blue) and *Myrmica rubra* (Myrmicinae, yellow; phylogenetic relationship between the three subfamilies indicated by sketched tree). We sampled one population per species in Italy (pop. 1), and two in Spain (pop. 2&3; geographic relationship given in the map). Populations 1, 2 and 3 refer to Orbetello, L’Escala and Sant Feliu de Guíxols for *Li. humile*; Volterra, L’Escala and Seva for *La. neglectus*; and Monza, Ripoll and Vilallonga de Ter for *M. rubra*, respectively. For each population, the viral diversity (i.e., the number of different viruses detected) is indicated by pie size, and the proportion of actively infecting viruses in population-specific color, as compared to only contaminating viruses (gray). Photocredit: Sina Metzler and Roland Ferrigato, ISTA.

We collected workers of the nonprotected ants, *Li. humile*, *La. neglectus* and *M. rubra* in April and May 2014. Each ant species was sampled from three different populations, two of which in relatively close geographic distance to one another in Spain and one, more distant to these, in Italy, resulting in a total of nine study populations (*Li. humile* from Orbetello, L’Escala and Sant Feliu de Guíxols; *La. neglectus* from Volterra, L’Escala and Seva; *M. rubra* from Monza, Ripoll and Vilallonga de Ter; Figure 1, details in Supplementary Table 2). After transport to the laboratory, we snap-froze ≥500 (mean 538, range 500–565) individuals per ant species and population in liquid nitrogen and stored the tubes at −80°C for further processing. Ant collection and all work in the laboratory followed European law and institutional guidelines.

### 2.2. RNA extraction

Before starting the extractions, we removed the poison gland reservoir from all *La. neglectus* ants by grabbing the acidopore with forceps and pulling the poison gland out, as the formic acid interferes with downstream applications. To obtain the highest RNA extraction efficiency possible, we processed ants in primary-pools optimized for ant number per species (5 ants each for *M. rubra* and *La. neglectus*, 10 ants for *Li. humile*). Primary-pools of ants were homogenized with 2 ceramic beads (2.8 mm) in 350 μL QIAzol Lysis Reagent (Qiagen) for 2 × 2min at 30 Hz in a TissueLyser II (Qiagen). After homogenization, an extra 400 μL QIAazol Lysis Reagent (Qiagen) was added along with 150 μL chloroform. The aqueous phase containing the total RNA was used for further RNA extraction following the standard protocol of the Qiagen miRNeasy 96 kit manual. We eluted in 45 μL H_2_O and ran the elution step twice with the same H_2_O to improve RNA concentration (although at the cost of RNA yield). Primary-pools were merged to generate a final pool of approximately 500–550 individuals (adults and brood) per site/species combination (resulting in 9 final pools; for details on sample compositions see Supplementary Table 2). RNA yield and quality of the final pools were measured on a Bioanalyzer (Agilent) and a Qubit Fluorometer (Invitrogen).

### 2.3. Sequencing

Pools were sent for cDNA library preparation and sequencing to Eurofins Genomics GmbH (Ebersberg, Germany). For each pool two libraries were sequenced: one library of ribosomal RNA depleted, random primed cDNA 150 bp paired-end on a HiSeq 2500 v3 in rapid run mode, and one library of PAGE size selected (19–30 bp) on a HiSeq 2000 v3 in an Illumina platform.

### 2.4. Bioinformatics of RNA-seq data

All paired-end RNA-seq reads (approx. 150 nucleotides (nt) length) were quality-controlled: adaptors were removed, low quality regions were trimmed and reads less than 36 (nt) of length were removed using Trimmomatic with default parameters (Bolger et al., 2014). The reads were *in silico* normalized to a maximum sequencing depth of 50 and contigs were assembled using Trinity v2.6.6 (Grabherr et al., 2011). Trinity seems to be sensitive to sequence variation, which is frequently encountered in virus sequence data, and therefore we extended the Trinity-contigs using CAP3 (Huang and Madan, 1999) and IVA (Iterative Virus Assembler) (Hunt et al., 2015). Contigs of at least 1,000 nt in length were used for BLASTX similarity search (Altschul et al., 1990) with an e-value threshold of 10^−10^ against RefSeq viral protein databases downloaded from the National Center for Biotechnology Information (NCBI Resource Coordinators, 2017) on May 15th 2018 to identify contigs of viral origin. Open reading frames (ORFs) were extracted for the potential virus contigs using NCBI’s ORF finder (NCBI Resource Coordinators, 2017) and a separate BLASTX similarity search was carried out with each of the ORFs. BWA-MEM (Li and Durbin, 2009) was used for mapping the clean RNA-seq reads against the annotated virus contigs to get RPKM counts (reads per kilobase per million mapped reads), which normalizes read counts for virus genome length and sample sequencing depth. We focused our analyses on RNA viruses since DNA viruses are typically much larger and more difficult to assemble reliably since not the whole genome is transcribed.

### 2.5. Bioinformatics of small RNA sequencing (sRNA-seq)

The single-end short-RNA sequencing (sRNA) reads were first trimmed of adapters and of reads less than 15 nt long using a perl script ‘sRNA_clean.pl’ included in the VirusDetect pipeline (Zheng et al., 2017). In addition, ribosomal RNAs (rRNA) were removed by aligning the reads against the SILVA rRNA database (Yilmaz et al., 2013) using Bowtie2 v2.3.4.2 (Langmead and Salzberg, 2012). The filtered reads were then used for virus identification using the VirusDetect pipeline (Zheng et al., 2017), which first maps the reads against known viruses. Here, we used the insect virus reference database ‘vrl_Invertebrates_220_U97’ downloaded from ftp://bioinfo.bti.cornell.edu/pub/program/VirusDetect/virus_database/v220/U97/. In the VirusDetect pipeline, the mapping was followed by a reference-guided assembly of reads that matched the database virus sequences. The unmapped sRNA reads were assembled *de novo* to obtain contigs that were used for BLASTN and BLASTX searches against virus nucleotide and protein databases, respectively, to describe novel viruses. Finally, the filtered sRNA reads were mapped against undetermined contigs from BLAST searches to obtain the siRNA and piRNA size distribution for each of the contigs to help in the discovery of potentially novel viruses (Zheng et al., 2017).

The filtered sRNA-seq reads were also mapped, allowing for one mismatch, against the newly assembled virus genomes using Bowtie (Langmead et al., 2009) keeping only reads that uniquely mapped to the genomes (mapping quality >20). A modified version of the R package ViRome (Watson et al., 2013) was used for visualizing the sRNA size distribution of the mapped reads.^1^ We excluded contigs from further analysis, which did not meet at least one of the two following criteria: (i) induction of the host RNAi response based on normalized sRNA-seq read count (RPKM) per contig being higher than 10, while the proportion of 21-22 nt siRNA being higher than 50%, and (ii) abundance, whereby the normalized RNA-seq read count (RPKM) per virus genome was higher than 10, to allow for detection of viruses that have evolved to evade the host RNAi response. 5’prime base preferences were tested for their significance by Chi Square tests against an equal distribution of the 4 bases (A,C,G,U; 4 × 2 contingency tables; VassarStats)^2^ and *p*-values adjusted for multiple testing by Bonferroni correction to obtain two-sided, adjusted *p*-values.

### 2.6. Phylogenetic analysis

Phylogeny reconstruction was carried out based on RNA-dependent RNA polymerase (RdRP) amino acid sequences of each virus and its nine best BLASTP hits. If the virus sequence matched several database viruses common to another virus of this study, their phylogenies were estimated jointly. In addition, a separate phylogeny consisting of only ant-derived virus RdRP sequences was reconstructed. Alignments were generated using E-INS-I method in MAFFT v7.313 (Katoh and Standley, 2013) and trimmed using trimAl v1.2 (Capella-Gutiérrez et al., 2009). Amino acid substitution models were selected by using ProtTest 3 (Darriba et al., 2011) and unrooted phylogenies were reconstructed using PhyML v.3.0 with Nearest Neighbor Interchange (NNI) tree topology search operation and approximate likelihood branch supports based on approximate Bayes method (Guindon et al., 2010).

## 3. Results

### 3.1. Description of 41 novel RNA viruses

**Figure 2:**
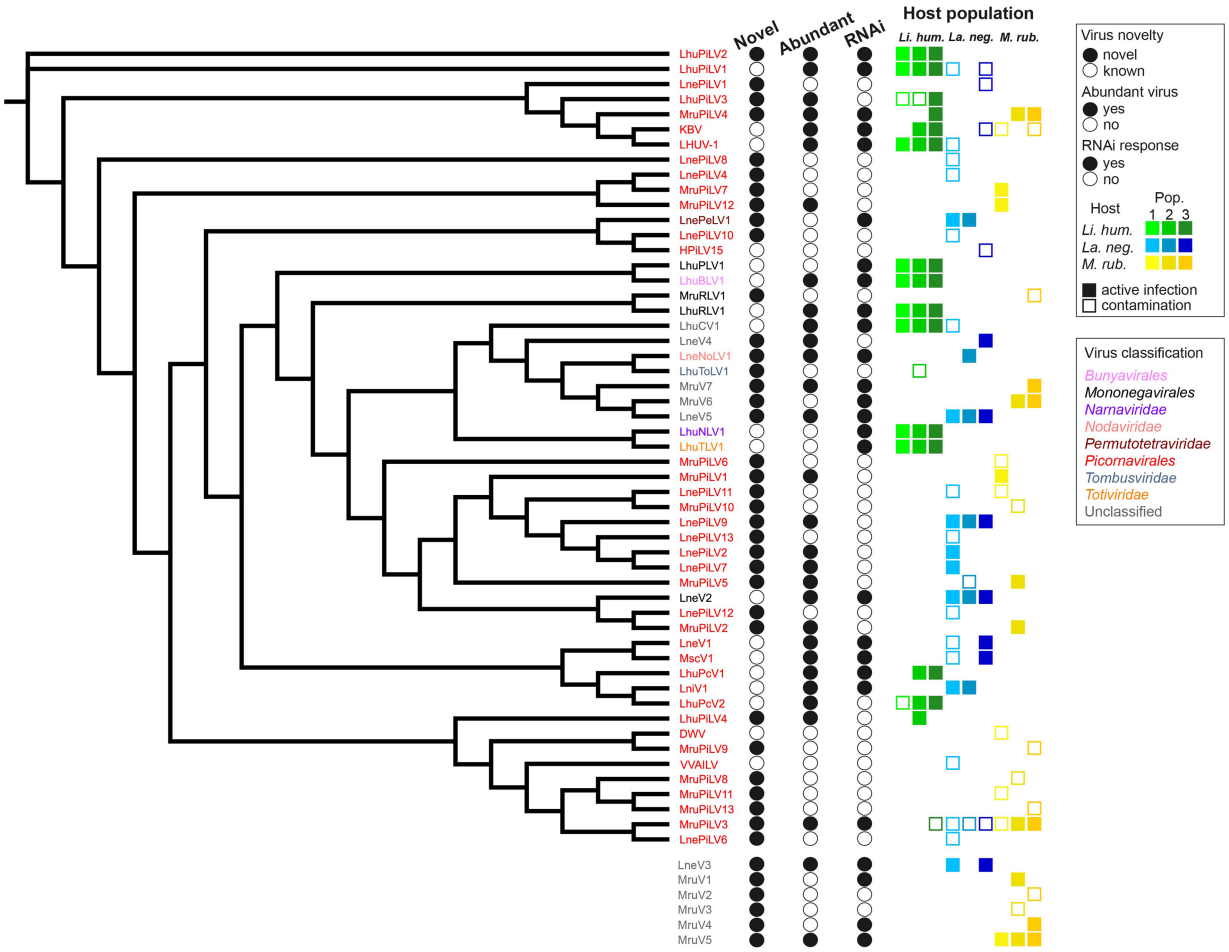
Virus dendrogram of the novel and known viruses. Dendrogram of the 53 viruses, for which we recovered the RdRP amino acid sequences (with the remaining 6 viruses, for which this sequence could not be determined shown below the dendrogram) that we detected in the three populations each of our three studied ant species (*Li. humile* in shades of green, *La. neglectus* in shades of blue, *M. rubra* in shades of yellow). Virus names are colored according to their phylogenetic classification. Filled or empty circles indicate whether the virus is newly described in this study or already known, if it is abundant in at least one of the studied populations (defined by the normalized RNA-seq read count [RPKM] that maps to the virus genome being higher than 10), and whether any of the host populations has raised an RNAi response against the virus (defined by sRNA-seq RPKM higher than 10 while the proportion of 21–22 nt siRNA is >50%). All the host populations, in which the virus was detected are shown for each virus as a square, as a filled square in the population-specific color (as in Figure 1), if consisting of an active infection, and an empty square where only representing a contamination (defined by the above abundance and siRNA criteria).

Our dual sequencing strategy of RNA-seq (150 nt reads) and sRNA-seq (19–30 nt reads) revealed a total of 59 viruses in our samples of the three study populations each from the ant species *La. neglectus*, *Li. humile*, and *M. rubra* (Table 1 and Figure 2). 31% (18/59) of these viruses were previously described based on Trinity-assembled contigs derived from the RNA-seq data (Table 1 and Figure 2). The remaining 69% (41/59, Supplementary Table 3) shared on average only 46% amino acid identity and an average query coverage of 67% (range 24–90% for identity and 9–99% for query coverage) with their best hit in BLASTX search. Most importantly, they shared less than 90% RdRP amino acid identity with their best hit in BLASTP search and are hence described as newly discovered viruses in this study (Edgar et al., 2022). For approximately half of these novel RNA viruses (21/41) we could reconstruct complete genomes, whilst the remaining (20/41) could only be recovered partially, either having one or two incomplete ORFs or missing essential genes such as those encoding for a capsid or an RNA-dependent RNA-polymerase (RdRP) (Supplementary Table 3). All these new virus identifications were based on the long sequence reads (RNA-seq). The sRNA-seq data, whilst essential for detecting the host antiviral response (see below), did not provide any additional information regarding novel virus sequences. The reason for this is that the assembly of the sRNA-seq reads to contigs yielded only fragmented information due to uneven distribution of the sRNA-seq reads on the virus genomes (for the mapping of the siRNA reads to the viral genomes that we obtained for both 14 known and 11 novel viruses see Supplementary Figure 1).

**Table 1:** Previously described and novel viruses discovered in this study.

	Virus name	Abbreviation	Classification	Genome size (bp)	Genome completeness	GenBank accession number
Known viruses	*Deformed wing virus*	DWV	*Picornavirales; Iflaviridae; Iflavirus*	4,562	Partial	MW314659
	*Hubei picorna-like virus 15*	HPiLV15	*Picornavirales*	4,281	Partial	MW314620
	***Kashmir bee virus variant LhuSP***	KBV_LhuSP	*Picornavirales; Dicistroviridae; Aparavirus*	9,567	Complete	MW314660
	***Linepithema humile bunya-like virus 1***	LhuBLV1	*Bunyavirales*	6,726	Complete	MW314661
	***Linepithema humile C-virus 1***	LhuCV1	Unclassified	2,482, 2,863	Complete	MW314662, MW314663
	***Linepithema humile narna-like virus 1***	LhuNLV1	*Narnaviridae*	2,187	Complete	MW314664
	***Linepithema humile partiti-like virus 1***	LhuPLV1	*Mononegavirales; Partitiviridae*	1701, 1,541	Complete	MW314670, MW314671
	***Linepithema humile picorna-like virus 1***	LhuPiLV1	*Picornavirales*	9,890	Complete	MW314667
	***Linepithema humile picorna-like virus 1 variant LhuSP1***	LhuPiLV1_LhuSP1	*Picornavirales*	9,293	Complete	MW314668
	***Linepithema humile picorna-like virus 1 variant LhuSP2***	LhuPiLV1_LhuSP2	*Picornavirales*	8,475	Partial	MW314669
	***Linepithema humile polycipivirus 1***	LhuPcV1	*Picornavirales; Polycipiviridae*	14,288	Complete	MW314665
	*Linepithema humile polycipivirus 2*	LhuPcV2	*Picornavirales; Polycipiviridae*	11,422	Complete	MW314666
	***Linepithema humile rhabdo-like virus 1***	LhuRLV1	*Mononegavirales; Rhabdoviridae*	12,087	Complete	MW314672
	***Linepithema humile toti-like virus 1***	LhuTLV1	*Totiviridae*	7,116	Complete	MW314673
	***Linepithema humile virus 1 variant LhuIT***	LHUV-1_LhuIT	*Picornavirales*	9,864	Complete	MW314674
	***Linepithema humile virus 1 variant LhuSP***	LHUV-1_LhuSP	*Picornavirales*	9,599	Complete	MW314675
	***Lasius neglectus virus 1 isolate LneSP***	LneV1_LneSP	*Picornavirales; Polycipiviridae; Sopolycivirus*	11,199	Partial	MW314676
	***Lasius neglectus virus 2 isolate LneSP***	LneV2_LneSP	*Mononegavirales; Rhabdoviridae*	12,164	Complete	MW314658
	***Lasius niger virus 1 variant LneITSP***	LniV1_LneITSP	*Picornavirales; Polycipiviridae; Sopolycivirus*	11,995	Complete	MW314677
	***Myrmica scabrinodis virus 1***	MscV1	*Picornavirales; Polycipiviridae; Sopolycivirus*	11,871	Complete	MW314678
	*Vespa velutina associated ifla-like virus*	VVAILV	*Picornavirales*	4,486	Partial	MW314619
Novel viruses	***Lasius neglectus noda-like virus 1***	LneNoLV1	*Nodaviridae*	2,944	Complete	MW314615
	***Lasius neglectus permutotetra-like virus 1***	LnePeLV1	*Permutotetraviridae*	4,848	Complete	MW314616
	*Lasius neglectus picorna-like virus 1*	LnePiLV1	*Picornavirales*	3,743	Partial	MW314618
	*Lasius neglectus picorna-like virus 2*	LnePiLV2	*Picornavirales*	6,666	Partial	MW314645
	*Lasius neglectus picorna-like virus 3*	LnePiLV3	*Picornavirales*	6,432	Complete	MW314626
	*Lasius neglectus picorna-like virus 4*	LnePiLV4	*Picornavirales*	4,141	Partial	MW314646
	*Lasius neglectus picorna-like virus 5*	LnePiLV5	*Picornavirales*	5,921	Partial	MW314650
	*Lasius neglectus picorna-like virus 6*	LnePiLV6	*Picornavirales*	6,658	Partial	MW314647
	*Lasius neglectus picorna-like virus 7*	LnePiLV7	*Picornavirales*	9,123	Partial	MW314623
	*Lasius neglectus picorna-like virus 8*	LnePiLV8	*Picornavirales*	4,745	Partial	MW314624
	*Lasius neglectus picorna-like virus 9*	LnePiLV9	*Picornavirales*	13,219	Complete	MW314625
	*Lasius neglectus picorna-like virus 10*	LnePiLV10	*Picornavirales*	5,688	Partial	MW314648
	*Lasius neglectus picorna-like virus 11*	LnePiLV11	*Picornavirales*	6,577	Partial	MW314649
	***Lasius neglectus virus 3***	LneV3	Unclassified	1,695	Partial	MW314617
	*Lasius neglectus virus 4*	LneV4	Unclassified	3,354, 3,060	Partial	MW314627, MW314628
	***Lasius neglectus virus 5***	LneV5	Unclassified	2,878	Complete	MW314622
	***Linepithema humile picorna-like virus 2***	LhuPiLV2	*Picornavirales*	9,092	Complete	MW314612
	*Linepithema humile picorna-like virus 3*	LhuPiLV3	*Picornavirales*	8,983	Complete	MW314613
	*Linepithema humile picorna-like virus 4*	LhuPiLV4	*Picornavirales*	6,073	Complete	MW314611
	*Linepithema humile tombus-like virus 1*	LhuToLV1	*Tombusviridae*	3,110	Complete	MW314614
	*Myrmica rubra picorna-like virus 1*	MruPiLV1	*Picornavirales*	9,630	Complete	MW314632
	*Myrmica rubra picorna-like virus 2*	MruPiLV2	*Picornavirales*	10,895	Complete	MW314633
	***Myrmica rubra picorna-like virus 3***	MruPiLV3	*Picornavirales*	10,215	Complete	MW314634
	*Myrmica rubra picorna-like virus 3 variant MruIT*	MruPiLV3_MruIT	*Picornavirales*	6,041	Partial	MW314657
	***Myrmica rubra picorna-like virus 4***	MruPiLV4	*Picornavirales*	10,003	Complete	MW314635
	*Myrmica rubra picorna-like virus 5*	MruPiLV5	*Picornavirales*	9,594	Complete	MW314636
	*Myrmica rubra picorna-like virus 6*	MruPiLV6	*Picornavirales*	5,539	Complete	MW314637
	*Myrmica rubra picorna-like virus 7*	MruPiLV7	*Picornavirales*	4,479	Partial	MW314654
	*Myrmica rubra picorna-like virus 8*	MruPiLV8	*Picornavirales*	5,451	Partial	MW314655
	*Myrmica rubra picorna-like virus 9*	MruPiLV9	*Picornavirales*	4,151	Partial	MW314652
	*Myrmica rubra picorna-like virus 10*	MruPiLV10	*Picornavirales*	4,186	Partial	MW314621
	*Myrmica rubra picorna-like virus 11*	MruPiLV11	*Picornavirales*	8,887	Complete	MW314629
	*Myrmica rubra picorna-like virus 12*	MruPiLV12	*Picornavirales*	5,396	Complete	MW314630
	*Myrmica rubra picorna-like virus 13*	MruPiLV13	*Picornavirales*	4,929	Complete	MW314631
	*Myrmica rubra rhabdo-like virus 1*	MruRLV1	*Rhabdoviridae*	5,010, 3,167	Partial	MW314639, MW314640
	***Myrmica rubra virus 1***	MruV1	Unclassified	1,243	Partial	MW314642
	*Myrmica rubra virus 2*	MruV2	Unclassified	4,731	Partial	MW314651
	*Myrmica rubra virus 3*	MruV3	Unclassified	5,417	Partial	MW314656
	***Myrmica rubra virus 4***	MruV4	Unclassified	1,198	Partial	MW314653
	***Myrmica rubra virus 5***	MruV5	Unclassified	1868	Partial	MW314638
	***Myrmica rubra virus 6 variant MruSP1***	MruV6_MruSP1	Unclassified	2,435	Complete	MW314641
	***Myrmica rubra virus 6 variant MruSP2***	MruV6_MruSP2	Unclassified	2,454	Complete	MW314644
	***Myrmica rubra virus 7***	MruV7	Unclassified	3,019	Complete	MW314643

Virus name, abbreviation used in this study and phylogenetic classification (to the degree of order, family or genus level depending on available information; unclassified in case the virus clustered with other unclassified sequences in the phylogeny or the virus genome assembly was missing RdRP) for all 59 viruses detected in this study. Note that some viruses were found as different variants (less than 95% similar at nucleotide level). Viruses exhibiting a positive RNAi response in bold. Genome size and completeness based on RNA sequencing (RNA-seq), with “complete” indicating that all the open reading frames in the assembled contig contained start and stop codons, whereas “partial” indicating that either the start or the stop codon was missing. GenBank accession number provided (see Supplementary Table 1 for references on previously published ant viruses).

### 3.2. Phylogeny of known and novel viruses

We had complete RdRP sequence information for 53 (out of the 59) viruses (16 known and 20 novel viruses with full genome information, as well as 15 out of 23 novel viruses with partial genome information). We first classified each of the 35 novel viruses with complete RdRP sequence separately by reconstructing a phylogeny together with its nine best BLASTP hits (Supplementary Figure 2). We then reconstructed a dendrogram of all our novel and known 53 viruses with complete RdRP sequence (Figure 2). Nearly ¾ of all the viruses identified in our study (38/53) were classified as picorna-like viruses, which have non-enveloped positive-sense single-stranded RNA genomes typically encoding one large polyprotein (Zell et al., 2017). There were also novel viruses belonging one each to the *Permutotetraviridae*, *Nodaviridae*, *Rhabdoviridae*, and *Tombusviridae* (Table 1). We were not able to classify 10 of the new virus sequences, as four of them clustered with unclassified viruses in the phylogeny (*La. neglectus:* LneV4 and LneV5, *M. rubra*: MruV6 and MruV7) and six could not even be included in the dendrogram due to their missing RdRP sequence (they were classified as viruses based on other identified virus proteins as follows: LneV3: coat/capsid protein; MruV1:capsid protein, MruV2: RNA helicase, MruV3: capsid, MruV4: capsid and MruV5: putative capsid).

### 3.3. 2/3 of the viruses cause active infections in the ants

To separate contaminants (i.e., viruses that are not infective to the ants but are, for instance, ingested with the food) from viruses that actively infect the ants we set two requirements, either of which needed to be met: as actively infecting viruses multiply in the host, they are expected to either reach high abundances and/or to be fought by the host immune system, resulting in virus-specific siRNA. Note that viral abundance and host RNAi response can be independent classifiers of an active infection as some highly replicating viruses may suppress the immune response (Li et al., 2002; Nayak et al., 2010; van Mierlo et al., 2012), or effective host siRNA prevents high viral loads from accumulating (Gammon and Mello, 2015). We defined a virus as (i) abundant, when the normalized RNA-seq read count (RPKM) that maps to the virus genome was higher than 10; and (ii) capable of inducing the RNAi response, when the sRNA-seq RPKM was higher than 10 while the proportion of 21–22 nt siRNA was higher than 50% (see methods; Supplementary Table 4). Using these criteria, we found that only 63% of the viruses in our study (37/59) cause active infections, whilst 37% are likely to only be contaminants (Supplementary Table 4). 22 of the 37 active viruses were novel and 15 were already known (Figure 2). Hence, from the 18 previously described viruses, only three (the deformed wing virus (DWV), a well-known bee virus (Wilfert et al., 2016; Martin and Brettell, 2019); the Hubei picorna-like virus 15 (HPiLV15), identified from a mix of arthropods (Shi et al., 2016); the Vespa velutina associated ifla-like virus (VVILV), identified from the invasive hornet *Vespa velutina* (Dalmon et al., 2019)) were neither abundant nor did they induce an RNAi response. Of the remaining 15, the majority were both abundant and induced the RNAi response, whilst only three caused a raised host immune response despite not being abundant. This shows that previous work applying classical long RNA-seq had already revealed many abundant and hence important viruses of ants. For the novel viruses, the picture was more diverse: 7/22 were both abundant and induced RNAi, 11/22 were only found to be abundant (e.g., LhuPiLV3, LnePiLV9, and MruPiLV4), and the remaining four caused an RNAi response but were not abundant (e.g., LnePeLV1 and MruV1). Hence, the latter could only be detected as an active infection based on their siRNA signature.

### 3.4. The RNAi response is host- and virus-specific

**Figure 3:**
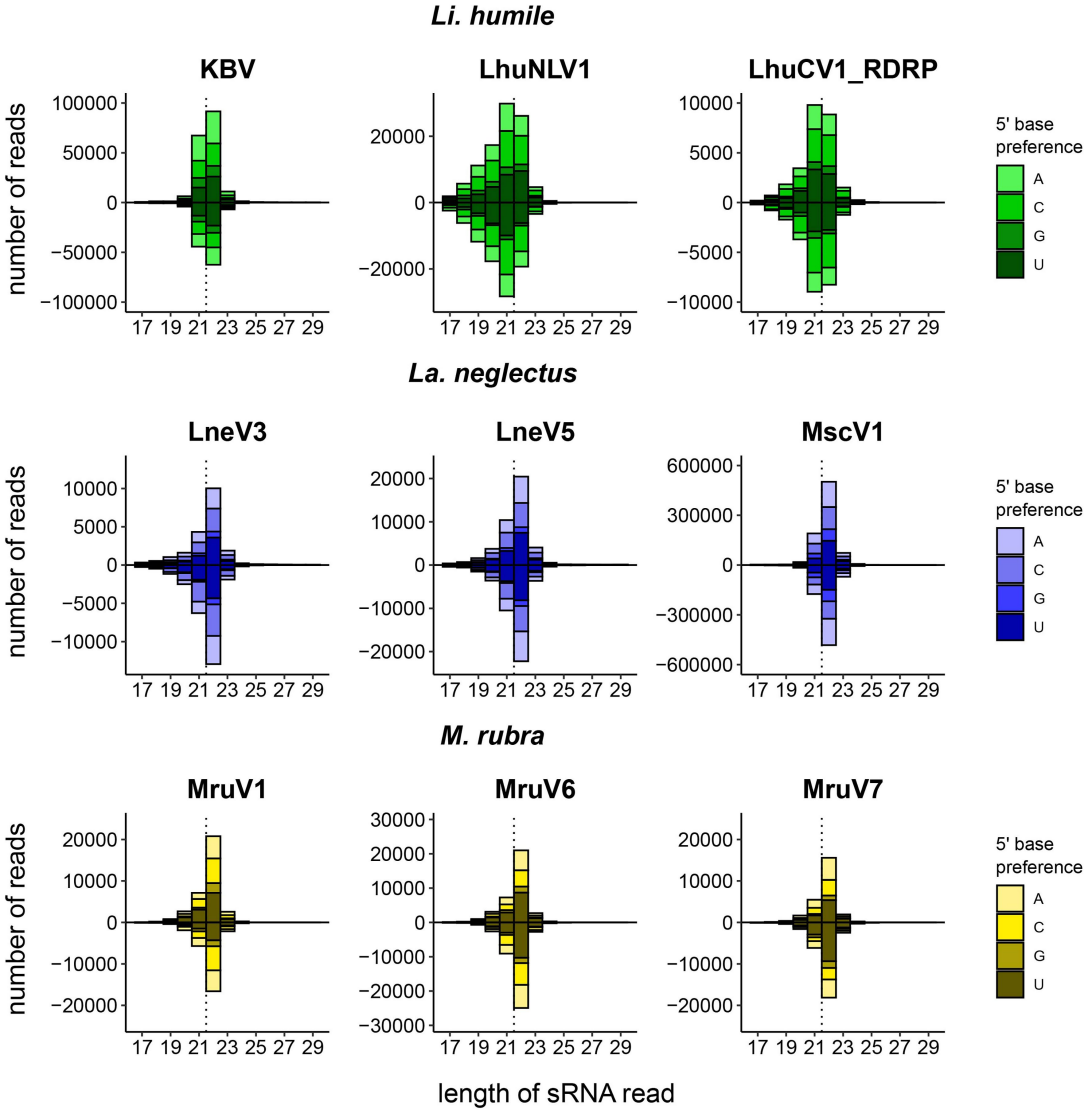
Host-dependent size distribution of the sRNA reads. For each ant species, *Li. humile* (green), *La. neglectus* (blue), and *M. rubra* (yellow), the three viruses with the highest number of sRNA reads are shown, separately for the positive strand (above the *x*-axis) and the negative strand (below the *x*-axis). In addition, the 5′-base is preference is given for each virus, showing that Guanine (G) was significantly underrepresented in positive and negative strands (ChiSquare tests; all *p*-values <0.002). Whilst *Li. humile* reacts with a virus-specific response of 21 or 22 nt (in 4 resp. 5 of the viruses, detailed in Supplementary Figure 3), both *La. neglectus* and *M. rubra* only produced 22 nt siRNA. Dotted vertical line separates 21 and 22 nt position. See Supplementary Figure 4 for four viruses with atypical sRNA-seq read size distribution.

We could detect a host RNAi response mostly against abundant viruses, as 72% (18/25) of the RNAi responses were found against abundant, and only 7/25 against non-abundant viruses. This confirms that high viral load is often predictive of an active infection, which then also triggers a host immune response. Our data reveal, however, the importance of direct measures of the host response, as nearly 30% of the viruses causing a siRNA response would otherwise have remained undetected. The size distribution of the small RNAs varied between species: *Li. humile* viruses showed distributions peaking at 21–22 nt with either 21 or 22 nt RNAs predominating depending on the virus (Supplementary Figure 3), whereas *La. neglectus* and *M. rubra* viruses showed distributions with a peak only at 22 nt (Figure 3). The size distributions of the small RNAs also revealed that sRNA reads map relatively equally to positive and negative strands of the viruses, and that guanine (G) was significantly underrepresented at 5′-base, similar to *D. melanogaster* (Figure 3; Webster et al., 2015). Moreover, in all ant species, we found one to two viruses that deviated from that general pattern. They either showed wide shoulders around major peaks at 21–22 nt in a low sRNA read count, even if their RNA-seq read count was high in abundance (LhuPcV2 in *Li. humile*; MruPiLV4 and MruPiLV5 in *M. rubra*), or they had both a very high RNA-seq and sRNA read count with the sRNA size distribution not indicating a clear overrepresentation of a certain length (LnePiLV9 in *La. neglectus*). These viruses also showed a bias toward the positive strand in the size distributions of sRNAs and a significant underrepresentation of G at 5′-base (Supplementary Figure 4).

**Figure 4:**
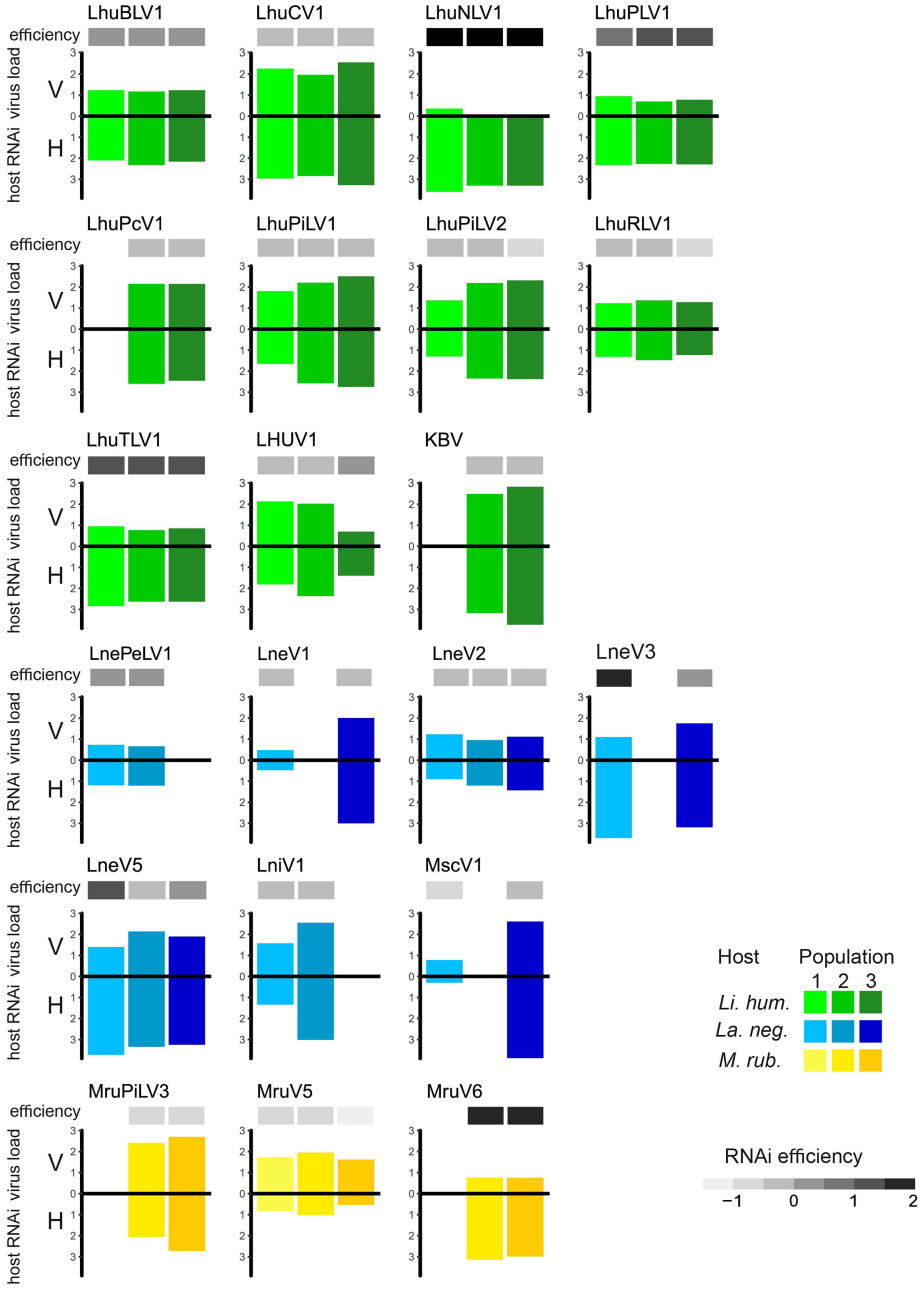
Efficiency of the host RNAi response. Virus load (V, measured as normalized RNA-seq read count [RPKM]; upper bar) and host RNAi response (H, measured as sRNA-seq RPKM; lower bar), as well as the resulting efficiency of the host RNAi response (ratio sRNA/viral load; the darker, the more efficient) is given for the 21 viruses that elicit an RNAi response in at least two study populations of the three ant species, *Li. humile* (in shades of green), *La. neglectus* (in shades of blue) and *M. rubra* (in shades of yellow); population-specific color-coding as detailed in Figure 1.

The efficiency of the host immune response, measured by the log-ratio of sRNA and RNA-seq RPKM values, showed considerable variation between viruses, indicating that some viruses generally elicit a higher RNAi response than others (such as LhuNLV1, LhuTLV1, and LhuPLV1 in *Li. humile* and MruV6 in *M. rubra*, Figure 4). Whilst the host response efficiency differed between viruses, it was highly consistent across host populations, which showed very little variation in their efficiencies toward the same viruses (Figure 4 and Supplementary Table 4). The only exceptions to this were observed for LneV3 and LneV5 in the Volterra population of *La*. *neglectus*, where the efficiency was markedly higher than in the other populations of *La. neglectus* against the same virus.

### 3.5. Viral infections differ in abundance and diversity between ant species

The overall virus abundance of the actively infecting viruses differed greatly between ant species: *Li. humile* had the highest number of virus-derived sequence reads based on RNA-seq (average normalized read count 1,168 RPKM in the three populations), followed by *La. neglectus* (760 RPKM) and *M. rubra* (541 RPKM) (Table 2). The virus abundancies also differed between populations within a species: for example, the load of the Orbetello population (458 RPKM) of *Li. humile* was only ¼ of that of the Sant Feliu de Guíxols population (1939 RPKM). In *La. neglectus* the highest load was found in L’Escala (935 RPKM) and the lowest in Volterra (434 RPKM). The Vilallonga de Ter population of *M. rubra* had the highest virus load (901 RPKM), Ripoll had markedly less (642 RPKM) and the Monza population the lowest (79 RPKM) (Table 2 and Supplementary Table 4). These differences were also reflected in the viral species diversity, both within and between populations of the ant species.

**Table 2:** Actively infecting viruses in the three studied ant species.

Known viruses	*Linepithema humile*						*Lasius neglectus*						*Myrmica rubra*					
	Italy		Spain				Italy		Spain				Italy		Spain			
	Orbetello		L’Escala		St Feliu d.Guíxols		Volterra		L’Escala		Seva		Monza		Ripoll		Vilallonga de Ter	
KBV_LhuSP			v	si	v	si												
LhuBLV1	v	si	v	si	v	si												
LhuCV1	v	si	v	si	v	si												
LhuNLV1		si		si		si												
LhuPcV1			v	si	v	si												
LhuPcV2			v		v													
LhuPiLV1	v	si			v	si												
LhuPiLV1_LhuSP1			v	si														
LhuPiLV1_LhuSP2			v	si														
LhuPLV1		si		si		si												
LhuRLV1	v	si	v	si	v	si												
LhuTLV1		si		si		si												
LHUV-1_LhuIT	v	si																
LHUV-1_LhuSP			v	si		si												
LneV1_LneSP											v	si						
LneV2_LneSP							v			si	v	si						
LniV1_LneITSP							v	si	v	si								
MscV1											v	si						
Novel viruses																		
LhuPiLV2	v	si	v	si	v	si												
LhuPiLV3					v													
LhuPiLV4			v															
LneNoLV1									v	si								
LnePeLV1								si		si								
LnePiLV2							v											
LnePiLV7							v											
LnePiLV9							v	si	v	si	v							
LneV3							v	si			v	si						
LneV4											v							
LneV5							v	si	v	si	v	si						
MruPiLV1													v					
MruPiLV2															v			
MruPiLV3															v	si	v	si
MruPiLV4						si									v		v	
MruPiLV5															v			
MruPiLV12													v					
MruV1																si		
MruV4																		si
MruV5													v		v	si	v	
MruV6_MruSP1																si		
MruV6_MruSP2																		si
MruV7																	v	si
**Virus-derived reads (RPKM)**	**458**		**1,108**		**1939**		**434**		**935**		**911**		**79**		**642**		**901**	
**Total no. of viruses / population**	**9**		**13**		**14**		**8**		**6**		**7**		**3**		**7**		**6**	
**Total no. of viruses / species**	**15**						**12**						**11**					

Abundant presence of viral sequences and presence of detectable levels of siRNA for the 37 actively infecting viruses for each ant species and study population, split up for already known and novel viruses. We identified a virus as causing an active infection in the host, when one of the two criteria were fulfilled: (i) viral abundance >10 RPKM (based on RNA-seq, indicated by “v”), (ii) sRNA RPKM >10 and 50% of reads of sizes 21–22 nt (indicated by “si”). For some viruses, both criteria were fulfilled at the same time (indicated by dark gray), whilst others had either high RNA-seq (light gray) or siRNA data (intermediate gray). The total viral abundance per population (the sum of virus-derived reads; RPKM), as well as total number of viruses per population resp. species are given.

**Figure 5:**
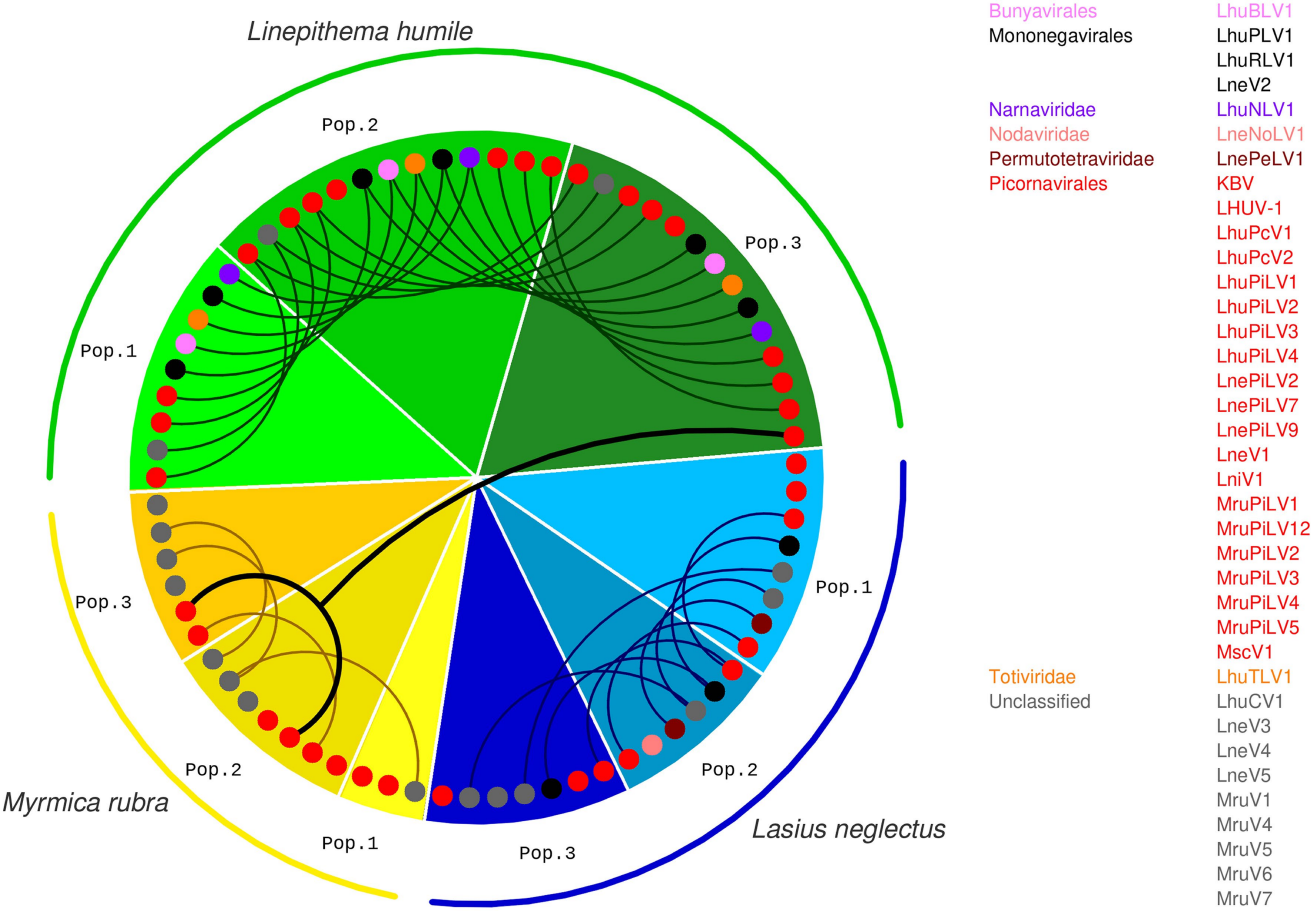
Presence and overlap of active viruses between ant species and populations. For each population of the three ant species, *Li. humile* (in shades of green), *La. neglectus* (in shades of blue) and *M. rubra* (in shades of yellow); color coding of ant populations and the phylogenetic position of the actively infecting viruses presented as in Figures 1, 2. Viruses occurring also in other populations are connected with a line, revealing a high, intermediate and low number of shared viruses between the populations of *Li. humile*, *La. neglectus* and *M. rubra*, respectively. Only a single virus (MruPiLV4) was shared between species, as it was found in two populations of *M. rubra* and one of *Li. humile*. Note that an interactive version of this figure is available under https://active-infections.science.ista.ac.at/.

The highest diversity of actively infecting viruses was discovered in *Li. humile*, which contained a total of 15 distinct active viruses with a high within-population variation of 9 to 14 viruses. In *La. neglectus,* we found intermediate diversity with a total of 12 viruses (6 to 8 per population), whilst *M. rubra* had the lowest within-population diversity with only 3 to 7 out of the total 11 viruses found in each of the three populations (Figure 2 and Table 2). We found the same pattern for between-population diversity: in *Li. humile,* 80% (12/15) of the viruses were shared between at least two, often even all three populations, whereas the proportion of shared viruses was only 50% (6/12) in *La. neglectus* and even only 36% (4/11) in *M. rubra* (Figure 5 and Table 2).

The number of different viral classes found in each species were in line with the viral abundance and diversity. The actively infecting viruses harbored by *Li. humile* represented eight different viral classes (*Bunyavirales, Dicistroviridae, Narnaviridae*, *Partitiviridae*, *Picornavirales, Polycipiviridae Rhabdoviridae*, *Totiviridae*) and one unclassified virus. *La. neglectus* contained five virus classes (*Nodaviridae, Permutotetraviridae, Picornavirales, Polycipiviridae, Rhabdoviridae*) and three unclassified viruses, whereas six of the viruses found in *M. rubra* belonged only to one class, the *Picornavirales*, and five were unclassified.

Overall, this revealed that *Li. humile* was most affected by active viral infections, both in terms of viral species diversity and their abundance, whilst *La. neglectus* was intermediate and *M. rubra* least infected.

### 3.6. Viral infections are specific to host species rather than region

Sampling each of our three ant species across a geographic area that overlapped between the three species allowed us to test if the viral infection patterns reflected host species-specificity or regional patterns. We clearly found that infective viruses were not geographically clustered but depended on host species across the sampled geographic range. Nearly all active virus infections were shared only between populations of the same ant species, but not across ant species, even if these were geographically very close in some sampling sites in Spain (Figures 1, 5). This host-specific infection pattern occurred even if the different populations of the same host species were quite distant from one another (max. linear distance >700 km between populations, such as between *La. neglectus* populations Volterra and Seva) (Figure 1). Across species, only one virus (MruPiLV4, Figure 2 and Table 2) was found to actively infect two of our ant species, namely one population of *Li. humile* and two populations of *M. rubra*, with the populations of the two species being 85 km apart (Figures 1, 5, Table 2, and Supplementary Tables 2, 4). In addition, six more viruses caused active infection in one host species, and were also detected in low amounts and not inducing an RNAi response in other species (empty squares in Figure 2), suggesting it occurred as a potential contaminant in these other ant species. In the two closest populations, *Li. humile* and *La. neglectus*, both from L’Escala, even only a single virus was shared, which was a contaminant only for both host ants (MruPiLV3; Figure 2). We therefore could not detect any regional clusters of either infective or contaminating viruses, but instead found that viral infection was very specific to our studied ant species.

## 4. Discussion

Viral infections of social insects have been studied extensively in bees (reviewed in Grozinger and Flenniken, 2019). In ants, however, only the invasive fire ant *Solenopsis invicta* was studied in detail (Valles et al., 2004, 2007; Hashimoto and Valles, 2008; Valles and Hashimoto, 2009; Valles and Rivers, 2019). We here used a novel dual approach for ants combining long and short RNA sequencing. This led to the description of 41 novel ant viruses, based on the long RNA-seq data. We also determined, which of the totally detected 59 viruses caused an active RNAi host response, based on the short sRNA reads mapping specifically to the viral genomes. Studying three populations each of three ant species representing the three major subfamilies of ants – *Linepithema humile*, Dolichoderinae; *Lasius neglectus*, Formicinae and *Myrmica rubra*, Myrmicinae – revealed host species-specific infection patterns with little regional or population-level effects, as well as host- and virus-specific efficiency of the RNAi response. Importantly, focusing on the actively infecting viruses – occurring either in high abundance or eliciting a detectable RNAi response – yielded different conclusions than a pure long-read study would have revealed. Notably, our study suggests very little overlap of active viral infections across ant species, respectively subfamilies. If we had instead drawn our conclusions solely on viral presence based on long RNA reads, as used in most previous work, this would have led to a seven-fold overestimate of cross-species sharing of virus infections.

Following the establishment of this dual approach by Webster et al. (2015) in *Drosophila*, we took advantage of insect hosts responding to viral infection by the RNAi response, leading to the production of 21–22 nt long siRNAs that can be uniquely mapped to the viral genomes and are indicative of an active host immune response. The peak of the size distribution of the virus-derived sRNAs is known to be different across insect species. For example, in bees the size distribution shows a peak at 22 nt (Chejanovsky et al., 2014), whereas in *Drosophila melanogaster* and Culicine mosquitoes the distribution peaks at 21 nt (Webster et al., 2015; Göertz et al., 2019). Here, we found that, in ants, there is no consistent peak size, but that the most abundant length depended both on the host ant and on the virus. Whilst the RNAi response in *La. neglectus* and *M. rubra* always peaked at 22 nt for all viruses, in *Li. humile* the most abundant lengths were both 21 and 22 nt, with the peak being either at 21 nt or at 22 nt, depending on the virus. Producing two major lengths of siRNAs within a single host species has also been observed for plants. In *Arabidopsis thaliana*, Dicer-like 4 (DCL4) produces 21 nt siRNAs and if DCL4 is suppressed by a viral suppressor of RNAi (VSR) then another enzyme (DCL2) is activated to produce 22 nt siRNAs (Deleris et al., 2006). Insect genomes typically encode for two Dicers, Dicer-1 and Dicer-2. In *Drosophila* the former has been associated with the generation of microRNAs that regulate host transcription, whereas only Dicer-2 is required for the production of siRNAs (Lee et al., 2004). The *Li. humile* genome also encodes for two Dicers, but it remains to be solved whether both are involved in the production of siRNAs, and whether this could explain the two sizes observed.

Applying this dual approach, we found 59 viruses in the natural populations of our three studied ant species, of which 18 were previously known RNA viruses and 41 were newly discovered by this study. Yet, only 63% (37/59) of these viruses caused active infections in the ants, using our criteria of either being highly abundant in their RNA-seq reads or eliciting an RNAi response. In approximately 50% of the cases, both criteria were fulfilled at the same time, whilst 32% (12/37) of the active viruses only showed high abundance and 19% (7/37) showed siRNA in the absence of a high viral load. This reveals that traditional approaches taking only viral abundance into account detect many important viruses but bear the risk of missing out on the ones that cause very efficient host response.

All three ants were able to raise an RNAi response, yet its effectiveness varied substantially against different viruses. Each ant species raised an extremely efficient response (as indicated by a high sRNA-seq/RNA-seq read count ratio) against several viruses (Figure 4). Whilst clearly infectious, these viral sequences can hence become too depleted to show high enough abundance to be picked up by long read analysis only. *Li. humile* and *La. neglectus* further contained three viruses that had both high viral load and high RNAi response, hence showing high viral replication despite the presence of a clearly raised host immune response. Interestingly, two of these viruses were first described in other species: KBV in the honeybee (Bailey and Woods, 1977; de Miranda et al., 2004) and LniV1 in the black garden ant *Lasius niger* (Olendraite et al., 2017).

On the other hand, some highly abundant viruses showed no detectable siRNA response, and four abundant viruses (LhuPcV2, LnePiLV9, MruPiLV4, and MruPiLV5) displayed atypical small RNA size distributions showing wide shoulders around the 22 nt peak (Supplementary Figure 4). Intriguingly, Drosophila C virus (DCV) and Drosophila Nora virus have a similar atypical small RNA size distribution (in their case with a peak at 21 nt in their *Drosophila* host) (Webster et al., 2015). Both DCV and Nora virus are known to encode a viral suppressor of RNAi (VSR) (van Rij et al., 2006; Lopez et al., 2018), raising the question whether the abnormal sRNA size distribution found for the four abundant viruses not eliciting a normal RNAi response in the host in our study might be a signature of a potent viral suppressor. Further support for these viruses encoding a VSR is their sRNA distributions displaying a skew toward the positive strand (Supplementary Figure 4), which is observed also in several other viruses encoding VSRs (Brackney et al., 2009; Han et al., 2011; Sabin et al., 2013). However, annotation of the ORFs of these ant viruses with similarity searches failed to find homologous proteins with such a function necessitating further research to evaluate whether any of the ORFs might indeed encode for a viral suppressor.

In our study, the number of different viruses per host species, including also the non-infective contaminant viruses, ranged from 17 to 29, which is in line with previous studies of viruses in arthropod hosts, e.g., 20 in the fruit fly *D. melanogaster* (Webster et al., 2015), 18 in the invasive fly *Drosophila suzukii* (Medd et al., 2018), 9 in the tick *Ixodes ricinus* (Pettersson et al., 2017), and 31 in the honeybee *Apis mellifera* (Remnant et al., 2017). Notably, the actively infecting viruses only reached much lower numbers of 3–14 viruses per ant population, and the number of viruses per population strongly differed between the three ant host species. *Li. humile* populations each contained a high number of viruses, whereas each *M. rubra* population was infected with much fewer viruses, with the Monza population of *M. rubra* containing only three infective viruses (Figures 2, 5). The viral clade diversity was also high in *Li. humile* and *La. neglectus*, whereas all identified viruses of *M. rubra* belonged to picorna-like viruses (Figures 2, 5). As all the ant species showed a clear RNAi response against most of the highly abundant viruses, these differences are unlikely to derive from biological differences in the ant species’ ability to defeat virus infections. Instead, a more plausible explanation could be the connectivity of colonies by the movement of individuals between nests within a population since connectivity is expected to promote virus transmission and thus increase virus abundance as well as diversity. This explanation is supported by the red fire ant *S. invicta*, where larger, interconnected multiple-queen colonies harbored higher viral diversity and load compared to smaller single-queen colonies (Valles et al., 2010; Allen et al., 2011; Brahma et al., 2021). Whereas *M. rubra* is native in Italy and Spain (Groden et al., 2005), the sampled *Li. humile* and *La. neglectus* populations are invasive (Giraud et al., 2002; Ugelvig et al., 2008), forming supercolonies consisting of large networks of aggression-free nests (Holway and Suarez, 1999), which could explain the more extensive virus sharing in populations of *Li. humile* and *La. neglectus* than in *M. rubra.*

Similar to their differences of within-population viral diversity, the three ants also showed different levels of sharing of their viruses between their populations. The three *Li. humile* populations had high, *La. neglectus* an intermediate and *M. rubra* only very little overlap of viruses between populations. This is in line with the non-invasive *M. rubra* populations being independently established by local queen swarming. The study populations of the two invasive species, on the other hand, more likely originated from non-independent introductions by human-mediated dispersal (Holway and Suarez, 1999; Giraud et al., 2002; Cremer et al., 2008). Whilst the origin of *La. neglectus* is still putative and its invasion history only partially resolved, we know that it is a relatively young invader that was only detected in Europe in the 1970s, and whose invasive populations have a high potential to spread to new places (Seifert, 2000; Ugelvig et al., 2008). The Argentine ant, on the other hand, has a much longer invasion history and has established massive invasions around the world including in Europe for 120 years (Suarez et al., 2001). Interestingly, for one of our study populations (Sant Feliu de Guíxols), we could perform an across-years comparison to viral samples collected 3 years earlier (Viljakainen et al., 2018). This revealed that all 11 previously described viruses were still present in the population, supporting the notion that these viruses establish long-term relationships with their hosts. Ten of these virus species have also been found in the Argentine ant in New Zealand (Gruber et al., 2017) and in California (Viljakainen et al., 2018) implicating that either the viruses originate from a historical infection predating the worldwide invasion of the Argentine ant or that these viruses are transmitted across continents.

Insect viruses are usually able to infect various host species (McMenamin et al., 2018). We hence tested how many of the viruses were shared between the three ant species. One of our *a priori* hypotheses was that, if viruses may be able to use several ant species as hosts, we could find some geographic patterns across species. We could not find support for a regional viral infection pattern, not even in Spain, where the three ant species co-occur in a small geographic area with distances between the populations ranging from being less than one to max. 84 km (Figure 1). This is also in line with new populations of the two invasive ants establishing *via* human-mediated jump dispersal rather than small-scale dispersal by flight like the native species (Holway and Suarez, 1999; Giraud et al., 2002; Cremer et al., 2008). Instead, we discovered that the great majority of viruses in this study caused infection in a host species-specific manner, i.e., their infectivity pattern was not shared between the three ant species studied nor described earlier in any other insect. We only found a single actively infecting virus to be shared between *Li. humile* and *M. rubra* (Figure 5), leading to a cross-species sharing of only 1.7% (1/59). It is noteworthy, that a conventional study approach based only on long RNA reads would have overestimated this value seven-fold, as 11.8% (7/59) of the viruses were shared between ant species. In all six additional cases, however, we found the virus to actively infect only one of the species, whilst not fulfilling the criteria in the other species. This suggests that viral infectivity may be strongly host-specific, whilst contamination occurs at a much higher frequency in ants.

Since the three study species belonged to three different subfamilies of ants, our resolution is not fine-grained enough to state whether the detected host specificity lies at the level of species or subfamilies. However, our data also suggest some cases of cross-infectivity between ant species and even subfamilies, as we found *La. neglectus* to be actively infected with LniV1 and MscV1, both described earlier from different ant species, either of the same genus (*Lasius niger*) or of a different subfamily (*Myrmica scabrinodis*, Myrmicinae) by Olendraite et al. (2017). As this study is based on RNA sequencing, it cannot be said for sure, however, if these viruses also caused active infections in the ants from which they were described. Moreover, *Li. humile* showed an active infection with the Kashmir bee virus (KBV) (Bailey and Woods, 1977; de Miranda et al., 2004) in this field study, which can also establish long term infections in laboratory colonies (Viljakainen et al., 2018). KBV is a well-known honeybee pathogen, that is also able to infect bumblebees (Singh et al., 2010) and wasps (Anderson, 1991). KBV has also been found to infect Argentine ants in New Zealand, where the viral load was markedly higher when the ant nests occurred close to honeybee hives (Gruber et al., 2017). This may also be true for some of the viruses that we have not identified as actively infecting in our study, maybe simply due to low prevalence among the pools of the 500 ants. One virus that has been shown to be a genuine ant-infecting virus is the Deformed Wing Virus (DWV), which has been observed in field-collected *M. rubra* at low levels, suggesting a spillover of this virus to ants, which is possible when ants nest close to beehives (Gruber et al., 2017; Schläppi et al., 2019). These observations support that some viruses can actively cross insect species barriers and actively infect different families (Formicidae and Apidae) within the order Hymenoptera, allowing for transmission from ant species to ant species or infection spillover from other hosts.

Viral transmission is particularly relevant in the light of some of these species being invasive pest species that may spread their diseases to the native ant fauna, similar to the well-described viral spillover from managed honeybees to the native bees and bumblebees (Fürst et al., 2014; McMahon et al., 2015). On the other hand, the potential exists for viruses to be used as effective biocontrol measures for invasive species (Oi et al., 2015, 2019; Valles et al., 2022), if their host specificity is narrow. Even if our study more than doubled the number of known ant viruses, we expect that our knowledge today represents only a minute fraction of the true viral diversity associated with the more than 15,000 ant species. To determine the specificity of infection and the transmission dynamics of viruses across the social insects, we advocate for more studies using the dual RNA-seq/sRNA-seq strategy to differentiate between active infections and non-disease-causing contaminations.

## Data availability statement

The datasets presented in this study can be found in online repositories. The names of the repository/repositories and accession number(s) can be found below: https://www.ncbi.nlm.nih.gov/, BioProject ID PRJNA681549; https://www.ncbi.nlm.nih.gov/genbank/, MW314611-MW314678.

## Author contributions

SC and MF designed the study. MF coordinated the ant collection. AG and MF prepared the samples for RNA extraction. AG performed the RNA extraction. MF and TR developed the sequencing details with Eurofins. LV performed the bioinformatic analysis with input from JJ, LT, TE, TR, and MF. LV, JO, TE, and SC conceived and prepared the figures with input from MF. LV and SC wrote the manuscript with input from TE, AG, MF, and TR. All authors contributed to the article and approved the submitted version.

## Funding

This study was funded by the Austrian Science Fund (FWF; M02076-B25 to MF) and the Academy of Finland (343022 to LV).

## Conflict of interest

The authors declare that the research was conducted in the absence of any commercial or financial relationships that could be construed as a potential conflict of interest.

## Publisher’s note

All claims expressed in this article are solely those of the authors and do not necessarily represent those of their affiliated organizations, or those of the publisher, the editors and the reviewers. Any product that may be evaluated in this article, or claim that may be made by its manufacturer, is not guaranteed or endorsed by the publisher.

## Footnotes

^1^ https://github.com/Edert/viRome_ggplot2

^2^ http://vassarstats.net/newcs.html
