# Supplementary material for: Antiviral immune response reveals host-specific virus infections in natural ant populations
Source: Front Microbiol. 2023 Mar 16;14:1119002. doi: 10.3389/fmicb.2023.1119002 (PMC10060816; doi:10.3389/fmicb.2023.1119002)
Supplement: Supplementary file 1 [file Data_Sheet_1.PDF]

Supplementary Material

for

Antiviral immune response reveals host-specific virus infections in natural ant populations

Lumi Viljakainen[§]*, Matthias A. Fürst[§], Anna V. Grasse, Jaana Jurvansuu, Jinook Oh, Lassi Tolonen, Thomas Eder, Thomas Rattei, Sylvia Cremer*

[§]) equal contribution
*) correspondence to lumi.viljakainen@oulu.fi; sylvia.cremer@ist.ac.at

1 Supplementary Figures and Tables

1.1 Supplementary Tables

Supplementary Table 1. RNA viruses discovered in ants prior to this study. List of viruses of ants published in 2004-2021. Columns provide information on the host ant species, the published virus name, and its phylogenetic classification (to the known level of order, family or genus). The last column gives reference to the publication that described the virus.

Host ant	Virus name	Classification[a]	Publication
Camponotus japonicus	*Wuhan ant virus*	*Mononegavirales; Rhabdoviridae*	Li et al., 2015
Colobopsis shohki	*Colobopsis shohki virus 1*	*Picornavirales; Polycipiviridae; Sopolycivirus*	Fukasawa et al., 2020
Formica fusca	*Formica fusca virus 1*	*Nyamiviridae*	Kleanthous, Olendraite, Lukhovitskaya, & Firth, 2019
F. exsecta	*Formica exsecta virus-1*	*Picornavirales; Dicistroviridae*	Dhaygude, Johansson, Kulmuni, & Sundström, 2019
F. exsecta	*Formica exsecta virus-2*	*Picornavirales; Iflaviridae*	Dhaygude et al., 2019
F. exsecta	*Formica exsecta* TSA	*Picornavirales; Polycipiviridae; Sopolycivirus*	Olendraite, Lukhovitskaya, Porter, Valles, & Firth, 2017
F. exsecta	*Formica exsecta virus-4*	*Mononegavirales*	Dhaygude et al., 2019
Linepithema humile	*Kashmir bee virus*	*Picornavirales; Dicistroviridae; Aparavirus*	Gruber et al., 2017; Viljakainen, Holmberg, Abril, & Jurvansuu, 2018
Li. humile	*Linepithema humile bunya-like virus 1*	*Bunyavirales*	Viljakainen et al., 2018
Li. humile	*Linepithema humile C-virus 1*	Unclassified	Viljakainen et al., 2018
Li. humile	*Linepithema humile narna-like virus 1*	*Narnaviridae*	Viljakainen et al., 2018
Li. humile	*Linepithema humile polycipivirus 1*	*Picornavirales; Polycipiviridae; Sopolycivirus*	Olendraite et al., 2017; Viljakainen et al., 2018
Li. humile	*Linepithema humile polycipivirus 2*	*Picornavirales; Polycipiviridae; Sopolycivirus*	Viljakainen et al., 2018
Li. humile	*Linepithema humile picorna-like virus 1*	*Picornavirales*	Viljakainen et al., 2018
Li. humile	*Linepithema humile partiti-like virus 1*	*Mononegavirales; Partitiviridae*	Viljakainen et al., 2018
Li. humile	*Linepithema humile rhabdo-like virus 1*	*Mononegavirales; Rhabdoviridae*	Viljakainen et al., 2018
Li. humile	*Linepithema humile toti-like virus 1*	*Totiviridae*	Viljakainen et al., 2018
Li. humile	*Linepithema humile virus 1*	*Picornavirales*	Gruber et al., 2017; Viljakainen et al., 2018
Lasius neglectus	*Lasius neglectus virus 1*	*Picornavirales; Polycipiviridae; Sopolycivirus*	Olendraite et al., 2017
La. neglectus	*Lasius neglectus* TSA	*Picornavirales; Polycipiviridae; Sopolycivirus*	Olendraite et al., 2017
La. neglectus	*Lasius neglectus virus 2*	*Mononegavirales; Rhabdoviridae*	Kleanthous et al., 2019
La. niger	*Lasius niger virus 1*	*Picornavirales; Polycipiviridae; Sopolycivirus*	Olendraite et al., 2017
Monomorium pharaonis	*Monomorium pharaonis* TSA	*Picornavirales; Polycipiviridae; Sopolycivirus*	Olendraite et al., 2017
Myrmica scabrinodis	*Myrmica scabrinodis virus 1*	*Picornavirales; Polycipiviridae; Sopolycivirus*	Olendraite et al., 2017
M. scabrinodis	*Myrmica scabrinodis virus 2*	*Picornavirales; Dicistroviridae*	Kleanthous et al., 2019
Nylanderia fulva	*Nylanderia fulva virus 1*	*Solinviviridae; Nyfulvavirus*	Valles et al., 2016
N. pubens	-	Three unnamed viruses	Valles, Oi, Yu, Tan, & Buss, 2012
Solenopsis invicta	*Solenopsis invicta virus 1*	*Picornavirales; Dicistroviridae; Aparavirus*	Valles et al., 2004
S. invicta	*Solenopsis invicta virus 2*	*Picornavirales; Polycipiviridae; Sopolycivirus*	Olendraite et al., 2017; Valles, Strong, & Hashimoto, 2007
S. invicta	*Solenopsis invicta virus 3*	*Solinviviridae*	Valles & Hashimoto, 2009
S. invicta	*Solenopsis invicta virus 4*	*Picornavirales; Polycipiviridae; Sopolycivirus*	Olendraite et al., 2017
S. invicta	*Solenopsis invicta virus 5*	*Picornavirales; Dicistroviridae*	Valles, Porter, & Calcaterra, 2018
S. invicta	*Solenopsis invicta virus 6*	*Picornavirales; Dicistroviridae; Cripavirus*	Valles & Rivers, 2019
S. invicta	*Solenopsis invicta virus 7*	Unclassified	Valles & Rivers, 2019
S. invicta	*Solenopsis invicta virus 8*	*Picornavirales; Polycipiviridae; Sopolycivirus*	Valles & Rivers, 2019
S. invicta	*Solenopsis invicta virus 9*	*Picornavirales; Dicistroviridae; Triatovirus*	Valles & Rivers, 2019
S. invicta	*Solenopsis invicta virus 10*	Unclassified	Valles & Rivers, 2019
S. invicta	*Solenopsis invicta virus 11*	*Picornavirales; Iflaviridae*	Valles & Rivers, 2019
S. invicta	*Solenopsis invicta virus 12*	*Picornavirales; Dicistroviridae; Aparavirus*	Valles & Rivers, 2019
S. invicta	*Solenopsis invicta virus 13*	*Picornavirales; Dicistroviridae; Aparavirus*	Valles & Rivers, 2019
S. invicta	*Solenopsis invicta virus 14*	*Bunyavirales*	Xavier, Allen, & Whitfield, 2021
S. invicta	*Solenopsis invicta virus 15*	*Mononegavirales*	Xavier, Allen, & Whitfield, 2021
S. invicta	*Solenopsis invicta virus 16*	*Picornavirales; Iflaviridae*	Xavier, Allen, & Whitfield, 2021
S. invicta	*Solenopsis invicta virus 17*	Unclassified	Xavier, Allen, & Whitfield, 2021
S. invicta	*Solenopsis midden virus*	*Totiviridae*	Valles & Rivers, 2019
Tapinoma melanocephalum	*Milolii virus*	Unclassified	Brettell, Mordecai, Pachori, & Martin, 2017

[a] presented in the order, family or genus level.

Supplementary Table 2. Collection sites and sample composition of the ants. For each population of the three ant species, the collection year, country, village/city and coordinates of the collection site are given. In addition, the sample composition, i.e. the number or workers, queens and brood items, that were pooled to obtain the sample, are provided.

Ant species	population	year	country	village/city	coordinates	Workers	Queens	Brood
Linepithema humile	1	2014	Italy	Orbetello	42°26'33.2"N 11°13'39.9"E	500		
Linepithema humile	2	2014	Spain	L'Escala	42°07'18.1"N 3°08'17.0"E	550		
Linepithema humile	3	2014	Spain	Sant Feliu de Guíxols	41°48'54.8"N 3°03'10.5"E	500		50
Lasius neglectus	1	2014	Italy	Volterra	43°24'00.0"N 10°51'00.0"E	405		107
Lasius neglectus	2	2014	Spain	L'Escala	42°07'18.1"N 3°08'17.0"E	550		
Lasius neglectus	3	2014	Spain	Seva	41°48'33.1"N 2°15'46.9"E	500		56
Myrmica rubra	1	2014	Italy	Monza	45°35'54.5"N 9°16'18.5"E	433	1	75
Myrmica rubra	2	2014	Spain	Ripoll	42°12' 3"N 2°11'26"E	527		22
Myrmica rubra	3	2014	Spain	Vilallonga de Ter	42°19'43.3"N 2°18'41.5"E	500		65

Supplementary Table 3. Novel viruses discovered in this study. The number of open reading frames (ORFs) and the encoded proteins for each virus are given based on NCBI's BLASTP and Conserved Domain searches. [a] denotes incomplete ORF at the 5'-end, [b] incomplete ORF at the 3'-end.

Virus Name	Abbreviation	No. of ORFs	Encoded proteins
Lasius neglectus noda-like virus 1	LneNoLV1	1	polyprotein (incl. methyltransferase and RNA dependent RNA polymerase (RdRP))
Lasius neglectus permutotetra-like virus 1	LnePeLV1	2	RdRP; capsid
Lasius neglectus picorna-like virus 1	LnePiLV1	1	polyprotein[a] (incl. peptidase and RdRP)
Lasius neglectus picorna-like virus 2	LnePiLV2	1	polyprotein[b] (incl. helicase and RdRP)
Lasius neglectus picorna-like virus 3	LnePiLV3	2	non-structural polyprotein (incl. RdRP); hypothetical protein
Lasius neglectus picorna-like virus 4	LnePiLV4	2	non-structural polyprotein[a] (incl. peptidase and RdRP); capsid[b]
Lasius neglectus picorna-like virus 5	LnePiLV5	2	polyprotein[a] (incl. RdRP and double-stranded RNA-binding domains (DSRM)); hypothetical protein
Lasius neglectus picorna-like virus 6	LnePiLV6	1	polyprotein[a] (incl. helicase and RdRP)
Lasius neglectus picorna-like virus 7	LnePiLV7	2	non-structural polyprotein (incl. helicase, ATPase, RdRP, DSRM); hypothetical protein[b]
Lasius neglectus picorna-like virus 8	LnePiLV8	4	RdRP[a]; capsid; hypothetical protein; hypothetical protein
Lasius neglectus picorna-like virus 9	LnePiLV9	2	polyprotein (incl. helicase, RdRP, DSRM); capsid
Lasius neglectus picorna-like virus 10	LnePiLV10	1	polyprotein[a, b] (incl. helicase, ATPase and RdRP)
Lasius neglectus picorna-like virus 11	LnePiLV11	1	polyprotein[a] (incl. RdRP and DSRM)
Lasius neglectus virus 3	LneV3	2	capsid; hypothetical protein
Lasius neglectus virus 4	LneV4	2	segment 1: polyprotein (incl. methyltransferase and RdRP). Segment 2: helicase
Lasius neglectus virus 5	LneV5	2	hypothetical protein; RdRP
Linepithema humile picorna-like virus 2	LhuPiLV2	2	polyprotein (incl. helicase and RdRP); capsid
Linepithema humile picorna-like virus 3	LhuPiLV3	2	polyprotein (incl. helicase, peptidase and RdRP); capsid
Linepithema humile picorna-like virus 4	LhuPiLV4	1	polyprotein (incl. helicase and RdRP)
Linepithema humile tombus-like virus 1	LhuToLV1	3	capsid; RdRP; hypothetical protein
Myrmica rubra picorna-like virus 1	MruPiLV1	1	polyprotein (incl. helicase and RdRP)
Myrmica rubra picorna-like virus 2	MruPiLV2	1	polyprotein (incl. ATPase, RdRP and DSRM)
Myrmica rubra picorna-like virus 3	MruPiLV3	1	polyprotein (incl. capsid, helicase, peptidase and RdRP)
Myrmica rubra picorna-like virus 3 variant MruIT	MruPiLV3_MruIT	1	polyprotein[a] (incl. helicase, peptidase and RdRP)
Myrmica rubra picorna-like virus 4	MruPiLV4	2	polyprotein (incl. helicase, peptidase and RdRP); capsid
Myrmica rubra picorna-like virus 5	MruPiLV5	1	polyprotein (incl. helicase and RdRP)
Myrmica rubra picorna-like virus 6	MruPiLV6	4	RdRP; hypothetical protein; capsid; capsid
Myrmica rubra picorna-like virus 7	MruPiLV7	3	RdRP[a]; capsid; capsid
Myrmica rubra picorna-like virus 8	MruPiLV8	1	polyprotein[a] (incl. helicase, peptidase and RdRP)
Myrmica rubra picorna-like virus 9	MruPiLV9	1	polyprotein[a] (incl. helicase, peptidase and RdRP)
Myrmica rubra picorna-like virus 10	MruPiLV10	1	polyprotein[a] (incl. RdRP and DSRM)
Myrmica rubra picorna-like virus 11	MruPiLV11	1	polyprotein (incl. capsid, helicase, peptidase and RdRP)
Myrmica rubra picorna-like virus 12	MruPiLV12	1	polyprotein (incl. helicase and RdRP)
Myrmica rubra picorna-like virus 13	MruPiLV13	1	polyprotein (incl. Helicase, peptidase and RdRP)
Myrmica rubra rhabdo-like virus 1	MruRLV1	5	segment 1: capsid; hypothetical protein; hypothetical protein; capsid. Segment 2[b]: RdRP
Myrmica rubra virus 1	MruV1	1	capsid
Myrmica rubra virus 2	MruV2	1	polyprotein[a, b] (incl. helicase)
Myrmica rubra virus 3	MruV3	1	capsid[b]
Myrmica rubra virus 4	MruV4	1	capsid[a]
Myrmica rubra virus 5	MruV5	1	hypothetical protein
Myrmica rubra virus 6 variant MruSP1	MruV6_MruSP1	2	hypothetical protein; RdRP
Myrmica rubra virus 6 variant MruSP2	MruV6_MruSP2	2	hypothetical protein; RdRP
Myrmica rubra virus 7	MruV7	2	RdRP; hypothetical protein

Supplementary Table 4. Virus abundance and host response. For each population of each of the three ant species, we provide details (sequence length, # mapped reads, RPKM, depth, fraction of viral reads) of the viral abundances (based on RNA-seq) and the triggered RNAi host response (based on sRNA-seq) for all actively infecting viruses (virus names in bold) and for potential contaminant viruses.

see Viljakainen_TableS4.xlsx

1.2 Supplementary Figures

Supplementary Figure 1

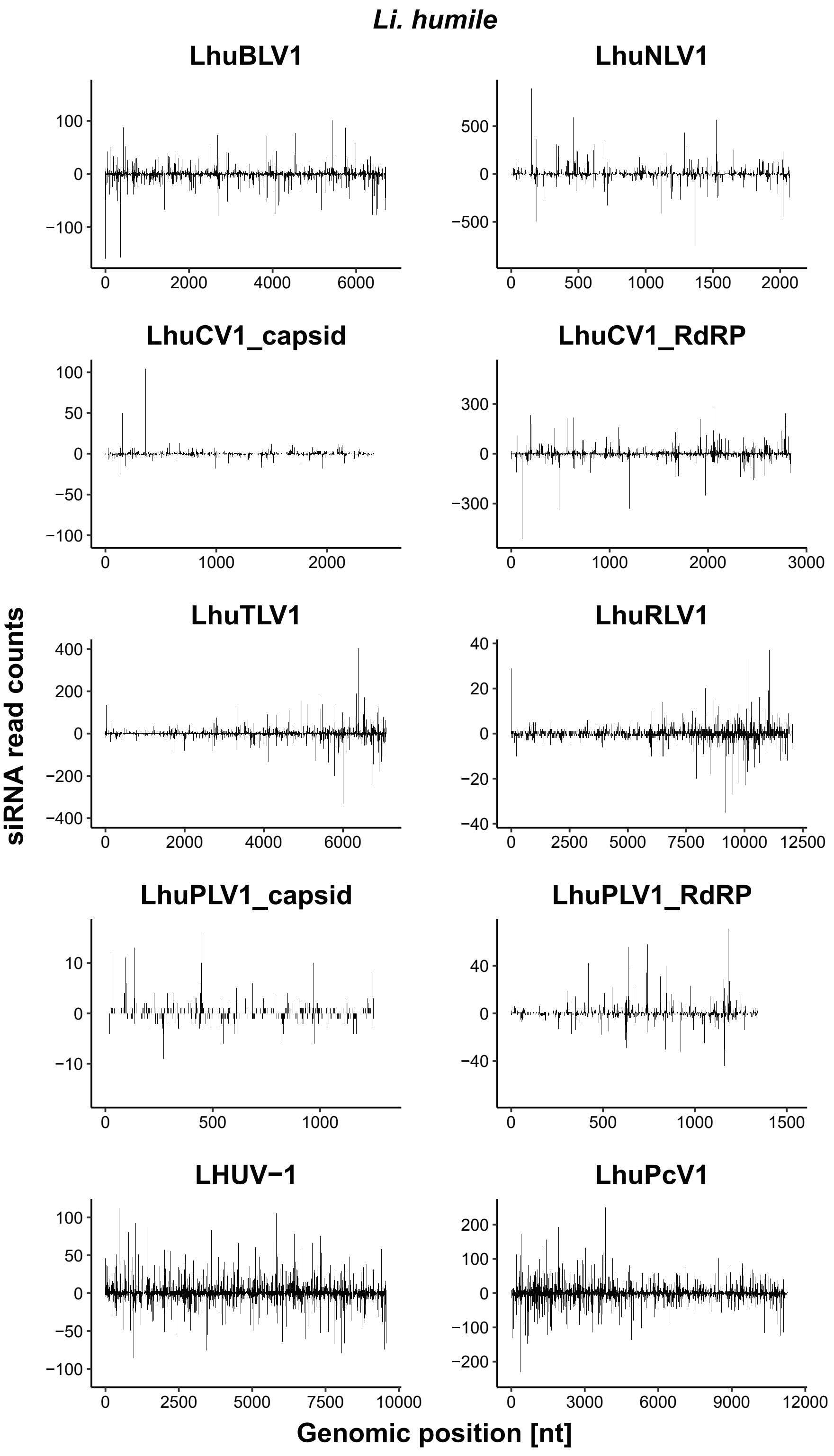

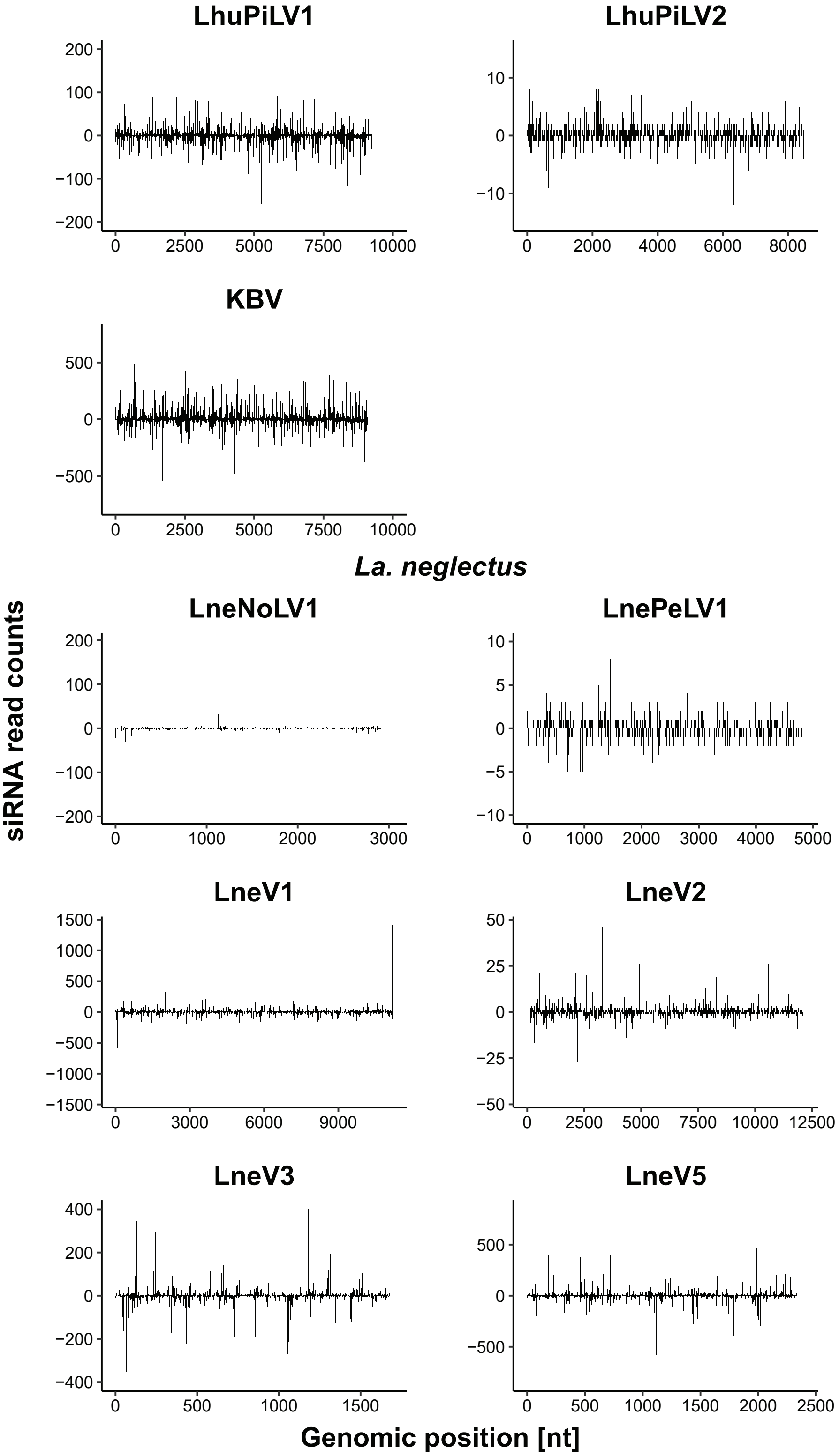

LhuPiLV1
LhuPiLV2
KBV
La. neglectus
LneNoLV1
LnePeLV1
LneV1
LneV2
LneV3
LneV5
siRNA read counts
Genomic position [nt]

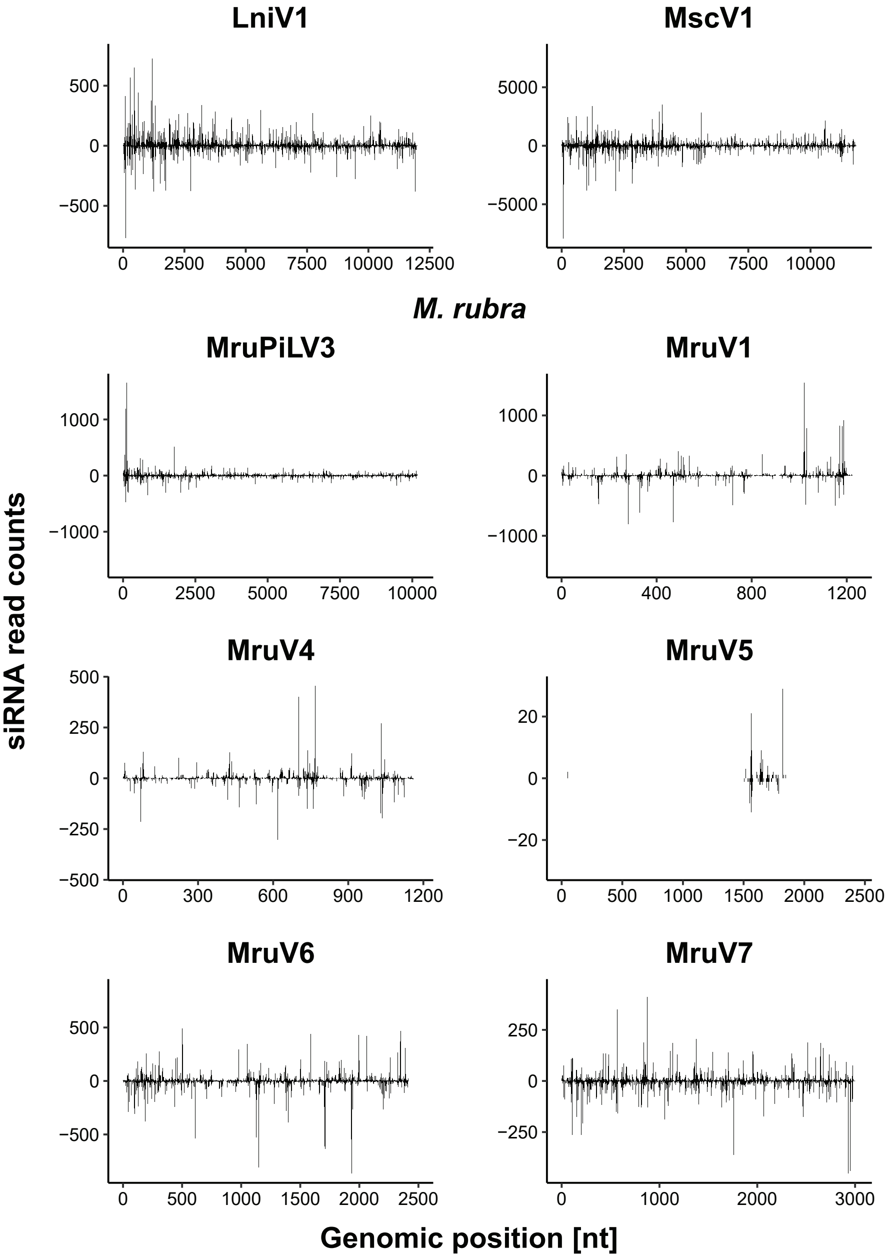

Supplementary Figure 1. Mapping of sRNAs to their viral genomes. Individual plots show the mapping of the small RNAs (sRNAs with confirmed proportion of 21-22nt being ≥ 50%) to their viral genomes assembled from the long RNA-seq data of the respective virus found in A) *Li. humile*, B) *La. neglectus* and C) *M. rubra* ants. All sequences are based on the complete virus genome assembly (two virus genomes, LhuCV1 and LhuPLV1 are bipartite). In total, 25 of the viruses (14 known and 11 novel) elicited a detectable RNAi response. For each virus, the x-axis shows the genomic position and y-axis the count of reads that map to each position. Reads that map to the positive strand are plotted above the x-axis and reads that map to the negative strand below the x-axis.

Supplementary Figure 2

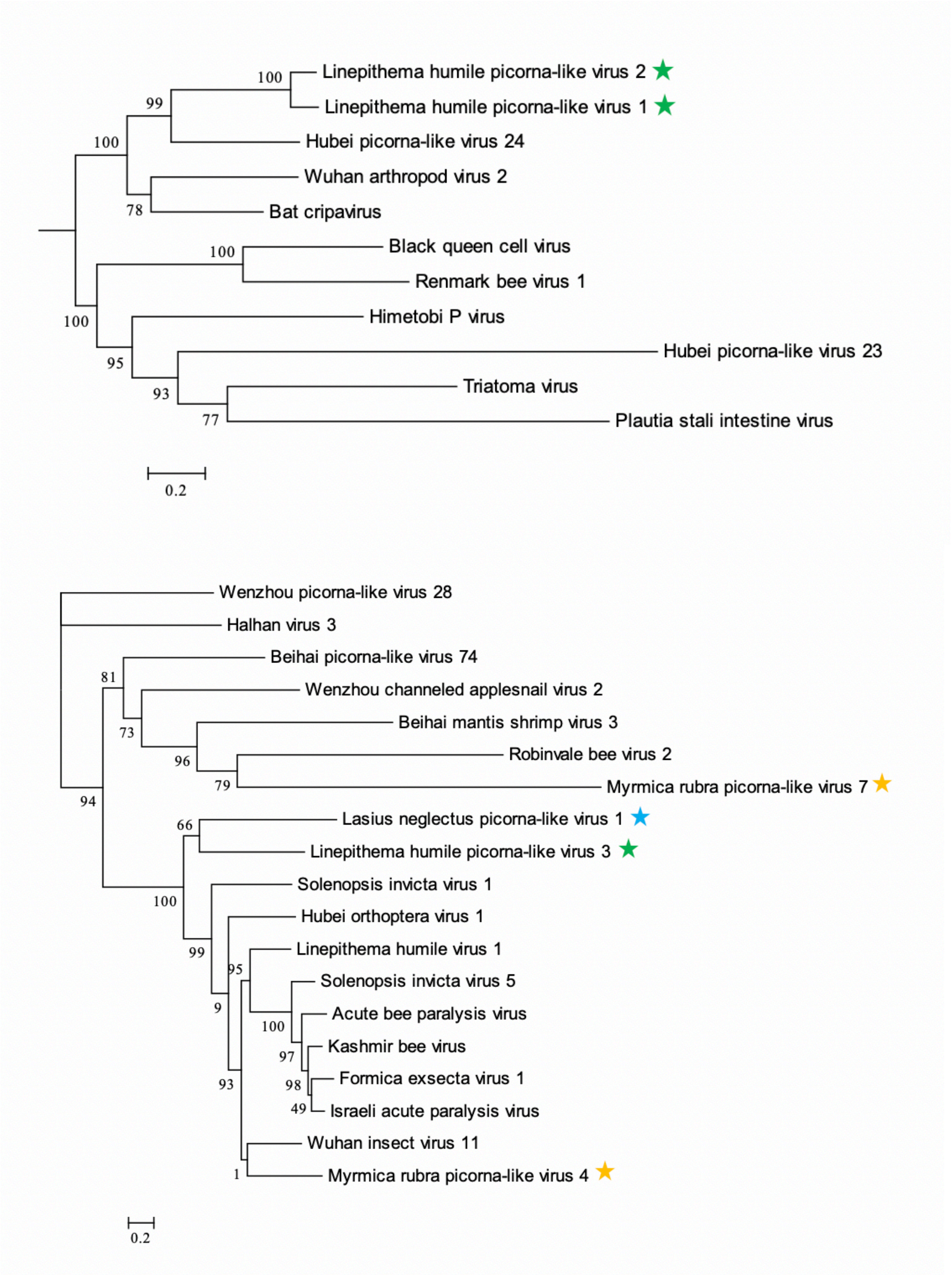

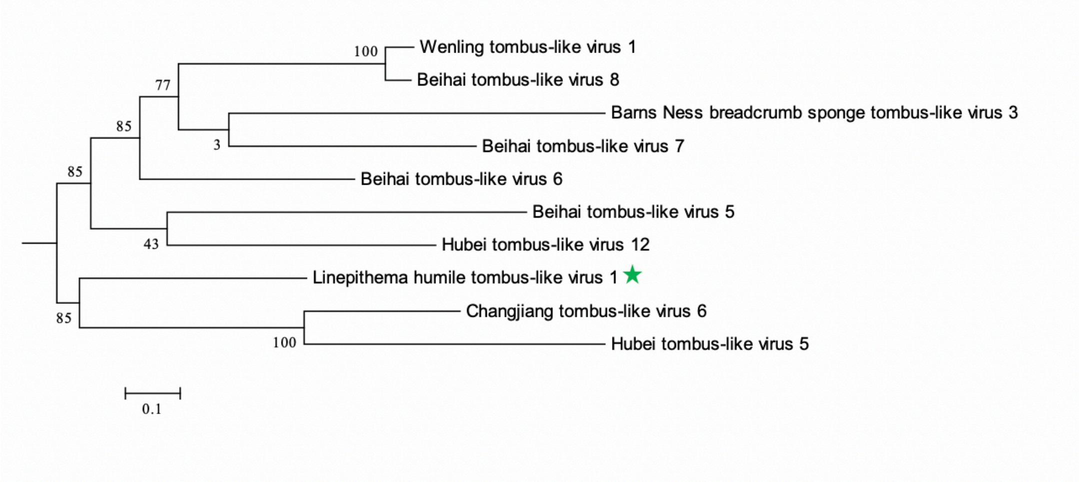
100
Wenling tombus-like virus 1
Beihai tombus-like virus 8
77
Barns Ness breadcrumb sponge tombus-like virus 3
85
3
Beihai tombus-like virus 7
85
Beihai tombus-like virus 6
Beihai tombus-like virus 5
43
Hubei tombus-like virus 12
Linepithema humile tombus-like virus 1
Changjiang tombus-like virus 6
85
100
Hubei tombus-like virus 5
0.1

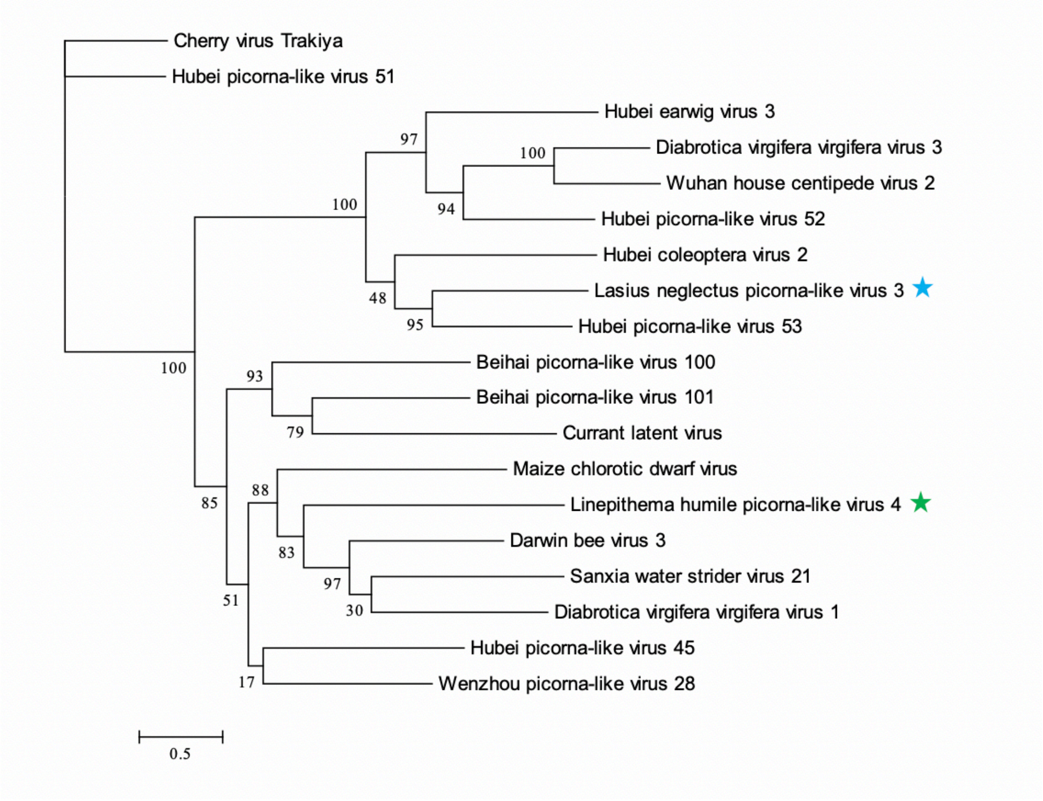
Cherry virus Trakiya
Hubei picorna-like virus 51
Hubei earwig virus 3
97
100
Diabrotica virgifera virgifera virus 3
Wuhan house centipede virus 2
100
94
Hubei picorna-like virus 52
Hubei coleoptera virus 2
Lasius neglectus picorna-like virus 3
48
95
Hubei picorna-like virus 53
Beihai picorna-like virus 100
100
93
Beihai picorna-like virus 101
79
Currant latent virus
Maize chlorotic dwarf virus
88
85
Linepithema humile picorna-like virus 4
Darwin bee virus 3
83
Sanxia water strider virus 21
97
51
30
Diabrotica virgifera virgifera virus 1
Hubei picorna-like virus 45
17
Wenzhou picorna-like virus 28
0.5

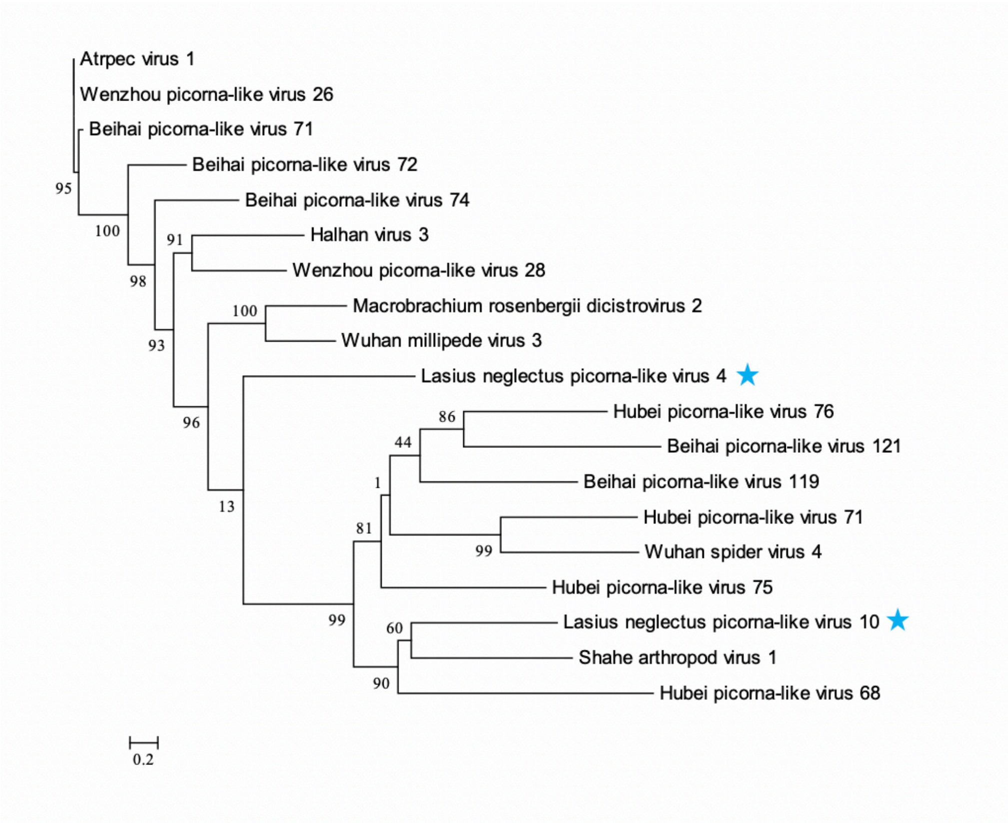
Atrpec virus 1
Wenzhou picorna-like virus 26
Beihai picorna-like virus 71
Beihai picorna-like virus 72
Beihai picorna-like virus 74
Halhan virus 3
Wenzhou picorna-like virus 28
Macrobrachium rosenbergii dicistrovirus 2
Wuhan millipede virus 3
Lasius neglectus picorna-like virus 4
Hubei picorna-like virus 76
Beihai picorna-like virus 121
Beihai picorna-like virus 119
Hubei picorna-like virus 71
Wuhan spider virus 4
Hubei picorna-like virus 75
Lasius neglectus picorna-like virus 10
Shahe arthropod virus 1
Hubei picorna-like virus 68
95
100
98
91
93
100
96
13
86
44
1
81
99
99
60
90
0.2

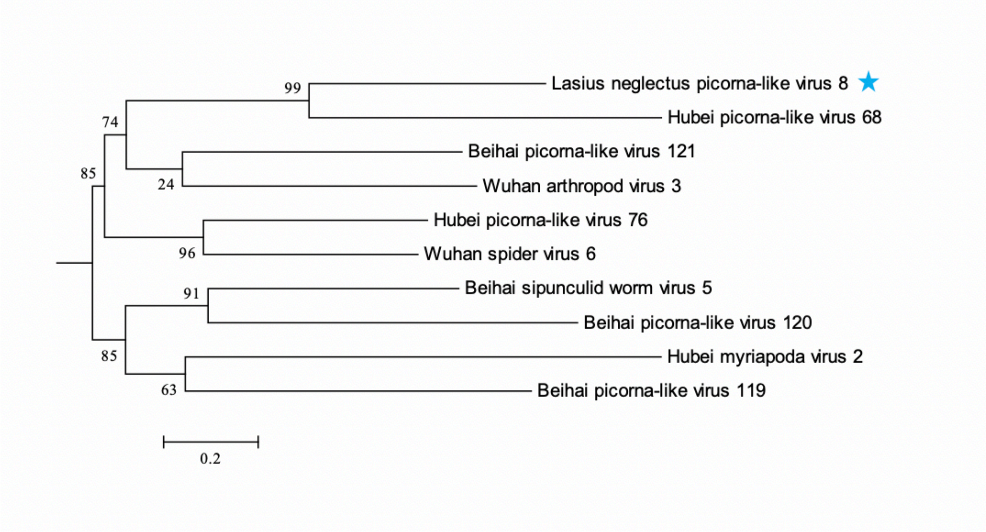
Lasius neglectus picorna-like virus 8
Hubei picorna-like virus 68
Beihai picorna-like virus 121
Wuhan arthropod virus 3
Hubei picorna-like virus 76
Wuhan spider virus 6
Beihai sipunculid worm virus 5
Beihai picorna-like virus 120
Hubei myriapoda virus 2
Beihai picorna-like virus 119
99
74
85
24
96
91
85
63
0.2

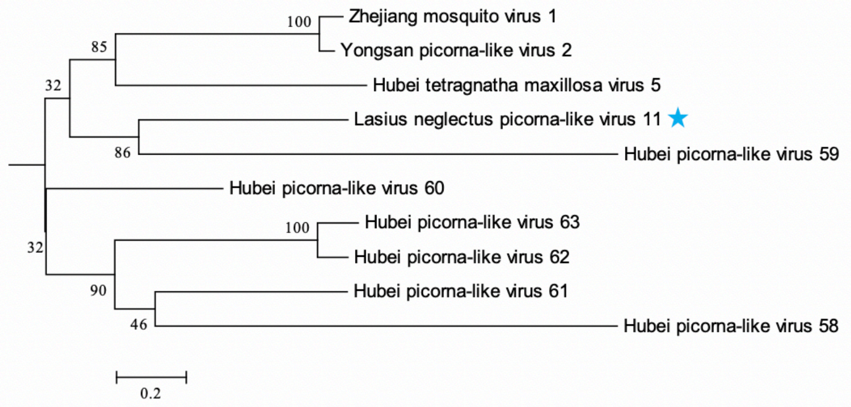
Zhejiang mosquito virus 1
Yongsan picorna-like virus 2
Hubei tetragnatha maxillosa virus 5
Lasius neglectus picorna-like virus 11
Hubei picorna-like virus 59
Hubei picorna-like virus 60
Hubei picorna-like virus 63
Hubei picorna-like virus 62
Hubei picorna-like virus 61
Hubei picorna-like virus 58
100
85
32
86
32
100
90
46
0.2

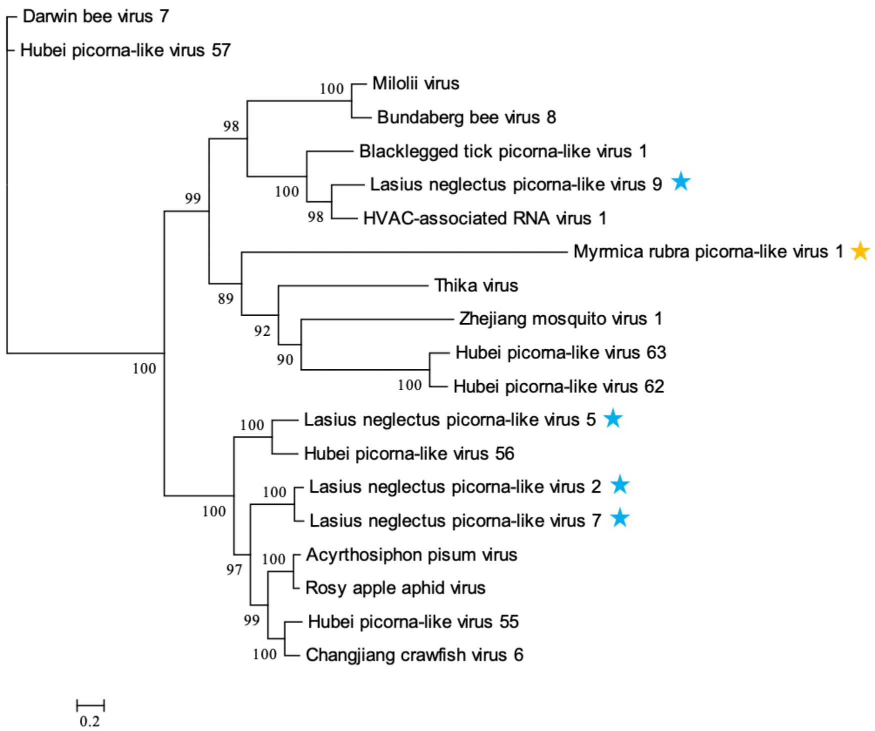
Darwin bee virus 7
Hubei picorna-like virus 57
Milolii virus
Bundaberg bee virus 8
Blacklegged tick picorna-like virus 1
Lasius neglectus picorna-like virus 9
HVAC-associated RNA virus 1
Myrmica rubra picorna-like virus 1
Thika virus
Zhejiang mosquito virus 1
Hubei picorna-like virus 63
Hubei picorna-like virus 62
Lasius neglectus picorna-like virus 5
Hubei picorna-like virus 56
Lasius neglectus picorna-like virus 2
Lasius neglectus picorna-like virus 7
Acyrthosiphon pisum virus
Rosy apple aphid virus
Hubei picorna-like virus 55
Changjiang crawfish virus 6
100
98
99
100
98
89
92
90
100
100
100
100
100
97
100
99
100
100
0.2

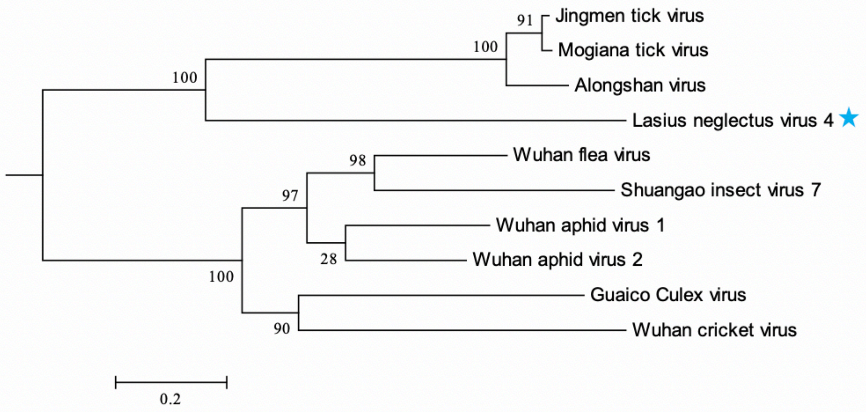
Jingmen tick virus
Mogiana tick virus
Alongshan virus
Lasius neglectus virus 4
Wuhan flea virus
Shuangao insect virus 7
Wuhan aphid virus 1
Wuhan aphid virus 2
Guaico Culex virus
Wuhan cricket virus
91
100
100
98
97
28
100
90
0.2

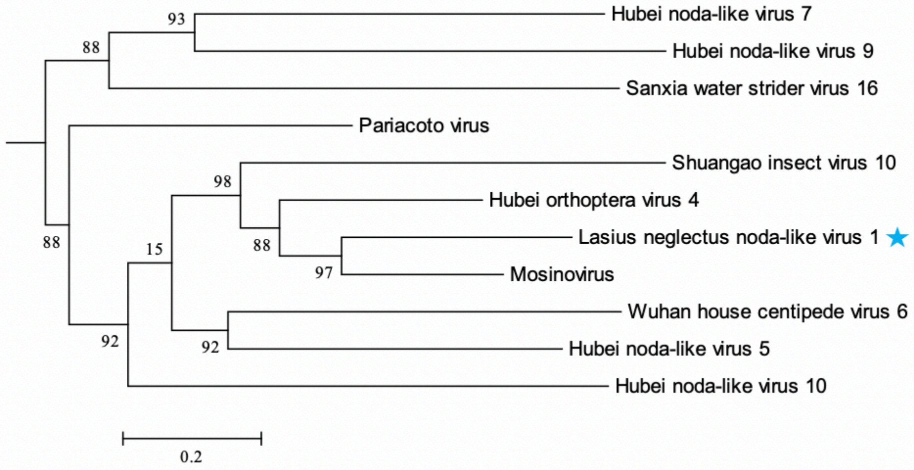
Hubei noda-like virus 7
Hubei noda-like virus 9
Sanxia water strider virus 16
Pariacoto virus
Shuangao insect virus 10
Hubei orthoptera virus 4
Lasius neglectus noda-like virus 1
Mosinovirus
Wuhan house centipede virus 6
Hubei noda-like virus 5
Hubei noda-like virus 10
93
88
98
88
15
88
97
92
92
0.2

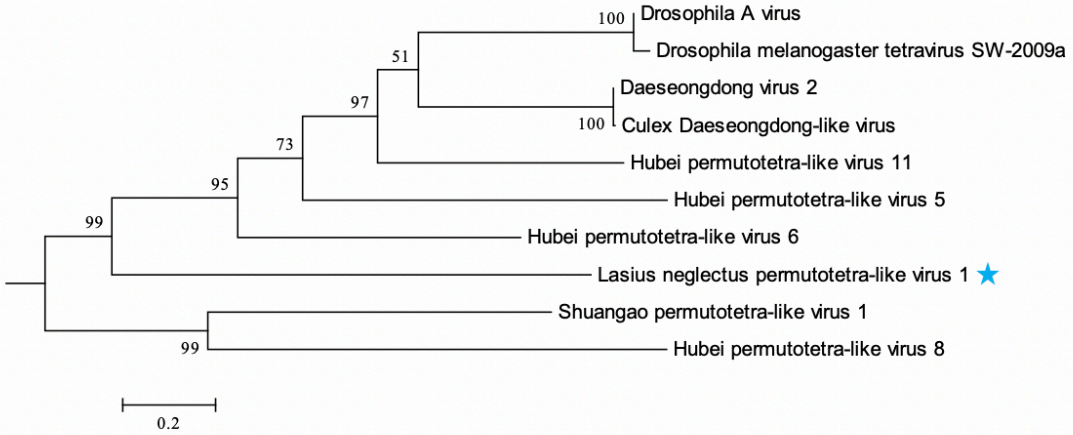
Drosophila A virus
Drosophila melanogaster tetravirus SW-2009a
Daeseongdong virus 2
Culex Daeseongdong-like virus
Hubei permutotetra-like virus 11
Hubei permutotetra-like virus 5
Hubei permutotetra-like virus 6
Lasius neglectus permutotetra-like virus 1
Shuangao permutotetra-like virus 1
Hubei permutotetra-like virus 8
100
51
97
100
73
95
99
99
0.2

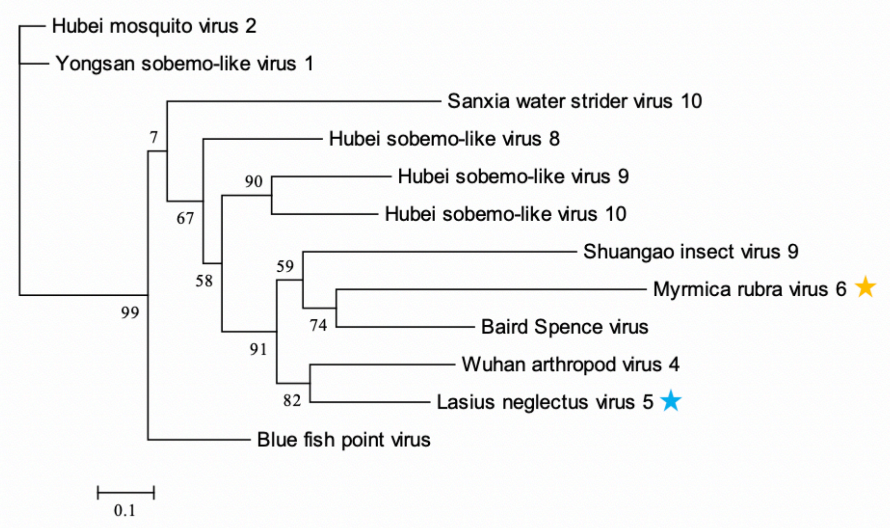
Hubei mosquito virus 2
Yongsan sobemo-like virus 1
Sanxia water strider virus 10
Hubei sobemo-like virus 8
Hubei sobemo-like virus 9
Hubei sobemo-like virus 10
Shuangao insect virus 9
Myrmica rubra virus 6
Baird Spence virus
Wuhan arthropod virus 4
Lasius neglectus virus 5
Blue fish point virus
7
90
67
59
58
74
99
91
82
0.1

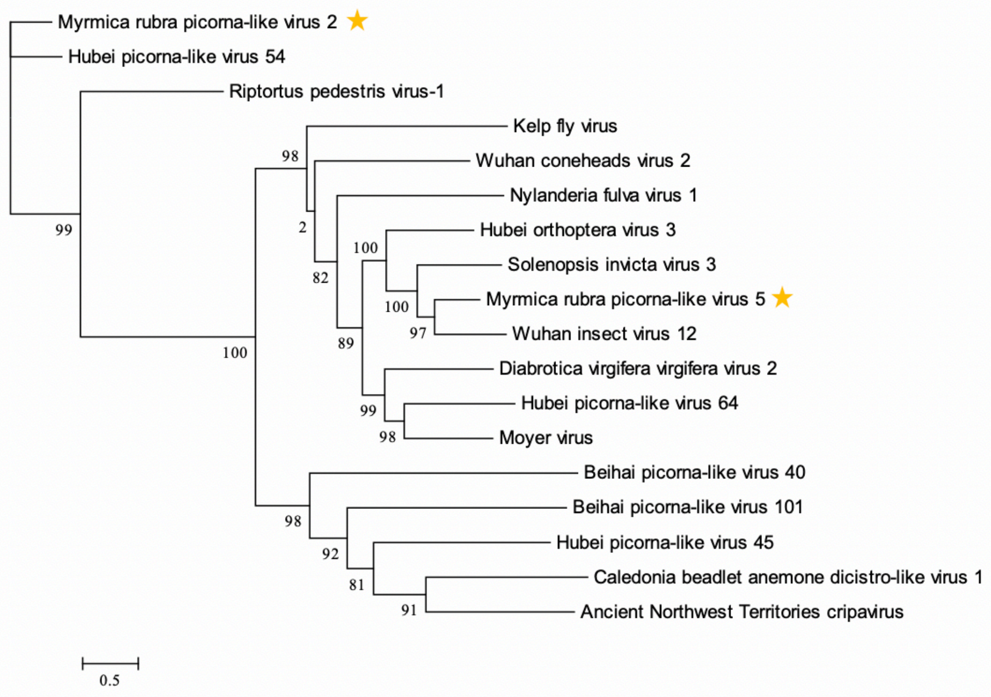
Myrmica rubra picorna-like virus 2
Hubei picorna-like virus 54
Riptortus pedestris virus-1
Kelp fly virus
Wuhan coneheads virus 2
Nylanderia fulva virus 1
Hubei orthoptera virus 3
Solenopsis invicta virus 3
Myrmica rubra picorna-like virus 5
Wuhan insect virus 12
Diabrotica virgifera virgifera virus 2
Hubei picorna-like virus 64
Moyer virus
Beihai picorna-like virus 40
Beihai picorna-like virus 101
Hubei picorna-like virus 45
Caledonia beadlet anemone dicistro-like virus 1
Ancient Northwest Territories cripavirus
98
99
2
100
82
100
97
89
100
99
98
98
92
81
91
0.5

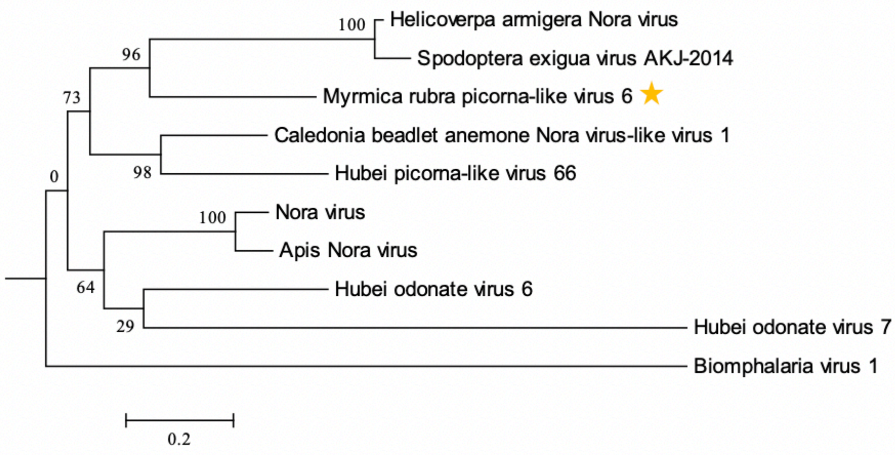

100
Helicoverpa armigera Nora virus
96
Spodoptera exigua virus AKJ-2014
73
Myrmica rubra picorna-like virus 6
Caledonia beadlet anemone Nora virus-like virus 1
0
98
Hubei picorna-like virus 66
100
Nora virus
Apis Nora virus
64
Hubei odonate virus 6
29
Hubei odonate virus 7
Biomphalaria virus 1
0.2

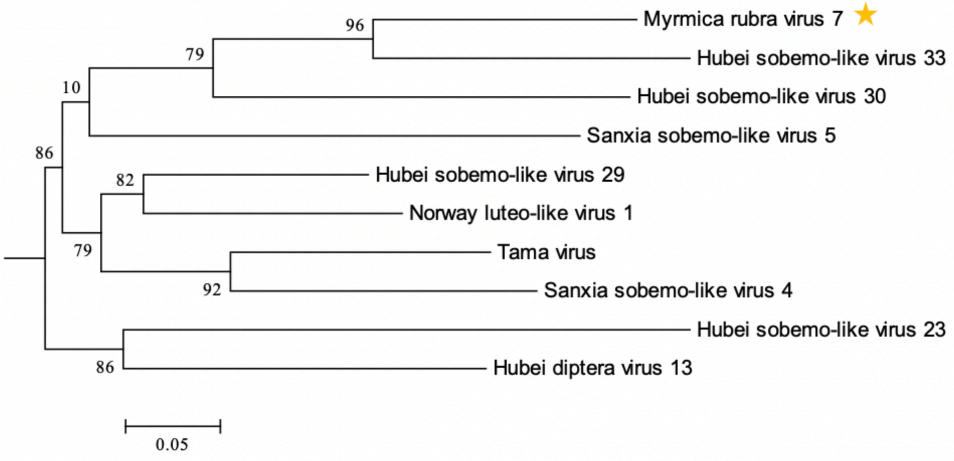

96
Myrmica rubra virus 7
79
Hubei sobemo-like virus 33
10
Hubei sobemo-like virus 30
Sanxia sobemo-like virus 5
86
82
Hubei sobemo-like virus 29
Norway luteo-like virus 1
79
Tama virus
92
Sanxia sobemo-like virus 4
Hubei sobemo-like virus 23
86
Hubei diptera virus 13
0.05

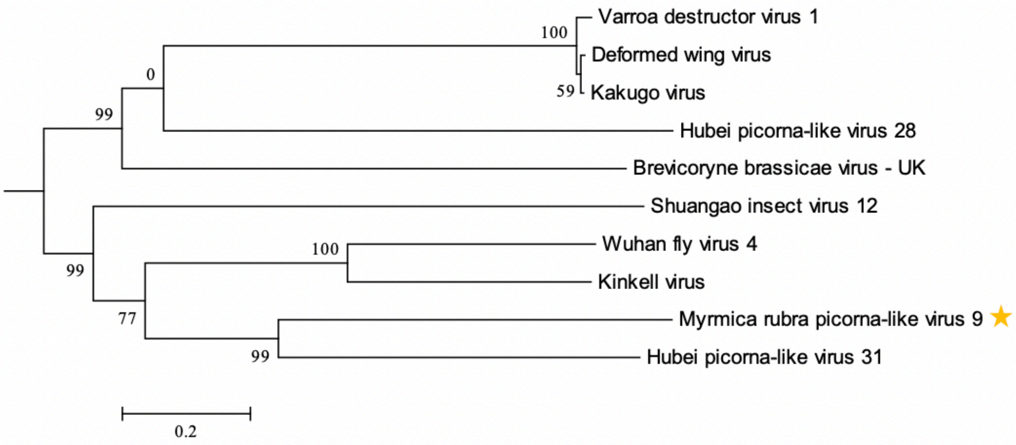

Varroa destructor virus 1
100
Deformed wing virus
0
59
Kakugo virus
99
Hubei picorna-like virus 28
Brevicoryne brassicae virus - UK
Shuangao insect virus 12
100
Wuhan fly virus 4
99
Kinkell virus
77
Myrmica rubra picorna-like virus 9
99
Hubei picorna-like virus 31
0.2

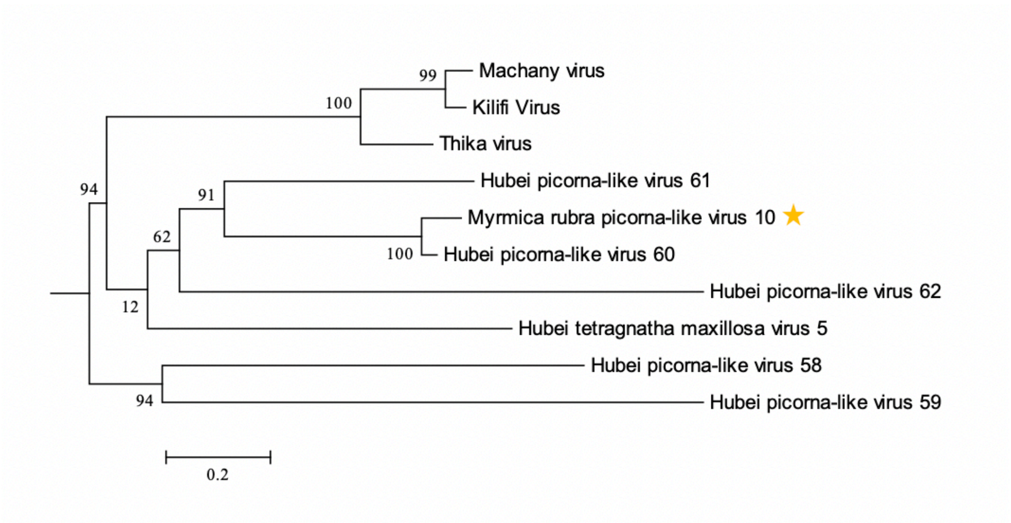
Machany virus
Kilifi Virus
Thika virus
Hubei picorna-like virus 61
Myrmica rubra picorna-like virus 10
Hubei picorna-like virus 60
Hubei picorna-like virus 62
Hubei tetragnatha maxillosa virus 5
Hubei picorna-like virus 58
Hubei picorna-like virus 59
99
100
94
91
62
100
12
94
0.2

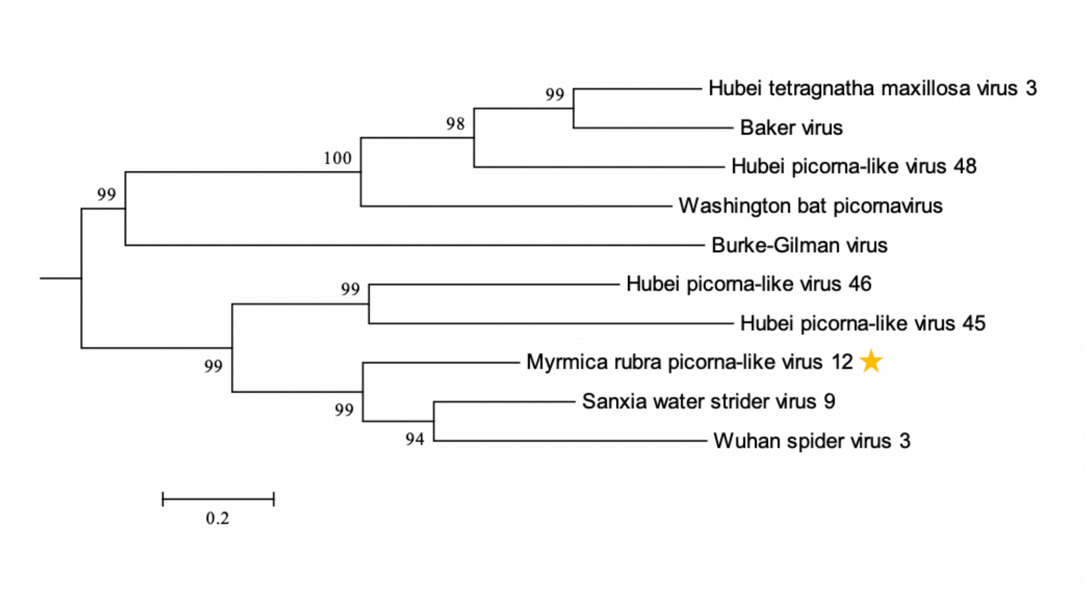
Hubei tetragnatha maxillosa virus 3
Baker virus
Hubei picorna-like virus 48
Washington bat picornavirus
Burke-Gilman virus
Hubei picorna-like virus 46
Hubei picorna-like virus 45
Myrmica rubra picorna-like virus 12
Sanxia water strider virus 9
Wuhan spider virus 3
99
98
100
99
99
99
99
94
0.2

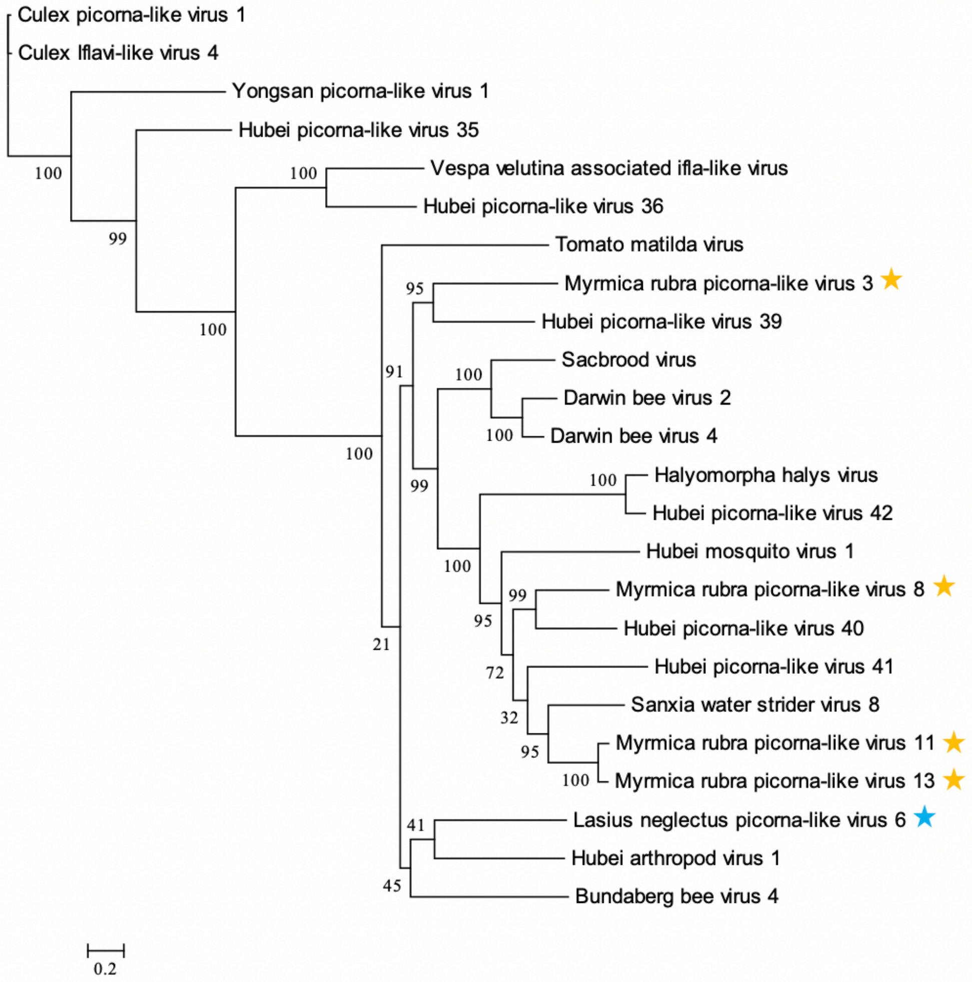
Culex picorna-like virus 1
Culex Iflavi-like virus 4
Yongsan picorna-like virus 1
Hubei picorna-like virus 35
Vespa velutina associated ifla-like virus
Hubei picorna-like virus 36
Tomato matilda virus
Myrmica rubra picorna-like virus 3
Hubei picorna-like virus 39
Sacbrood virus
Darwin bee virus 2
Darwin bee virus 4
Halyomorpha halys virus
Hubei picorna-like virus 42
Hubei mosquito virus 1
Myrmica rubra picorna-like virus 8
Hubei picorna-like virus 40
Hubei picorna-like virus 41
Sanxia water strider virus 8
Myrmica rubra picorna-like virus 11
Myrmica rubra picorna-like virus 13
Lasius neglectus picorna-like virus 6
Hubei arthropod virus 1
Bundaberg bee virus 4
100
99
100
100
100
95
91
100
100
99
100
100
99
95
21
72
32
95
100
41
45
0.2

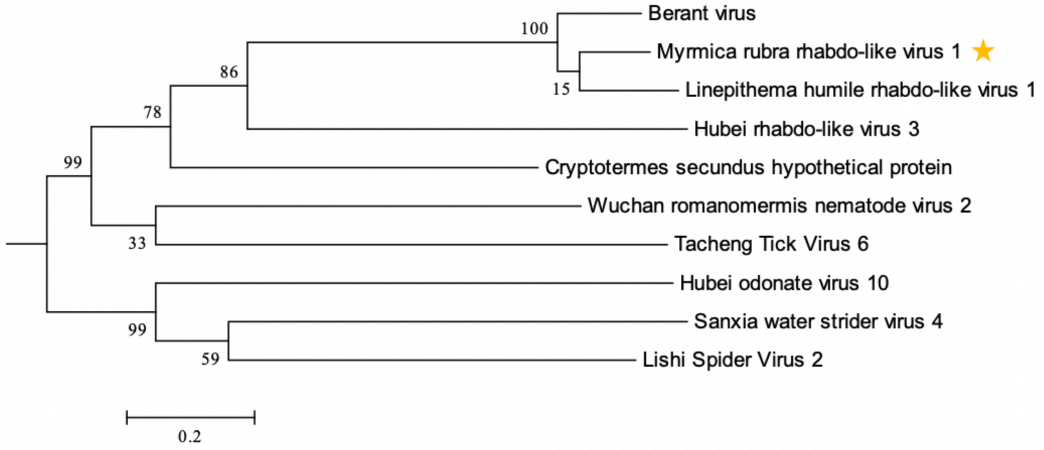
Berant virus
Myrmica rubra rhabdo-like virus 1
Linepithema humile rhabdo-like virus 1
Hubei rhabdo-like virus 3
Cryptotermes secundus hypothetical protein
Wuchan romanomermis nematode virus 2
Tacheng Tick Virus 6
Hubei odonate virus 10
Sanxia water strider virus 4
Lishi Spider Virus 2
100
15
86
78
99
33
99
59
0.2

Supplementary Figure 2. Phylogenies of the novel viruses found in this study. Phylogenetic trees for the novel viruses discovered in this study, based on RNA dependent RNA Polymerase amino acid sequences (RdRP) of each virus and its nine best BLASTP hits. If the virus sequence matched several database viruses common to another virus of this study, their phylogenies were estimated jointly. Viruses discovered in *Li. humile* are marked with green stars, in *La. neglectus* with blue stars and in *M. rubra* with yellow stars. The branch supports of the trees are posterior probabilities based on an approximate Bayes method in PhyML v. 3.0. The majority of the branches had high support, but some branches had low support, so that some phylogenies remained partially unresolved.

Supplementary Figure 3

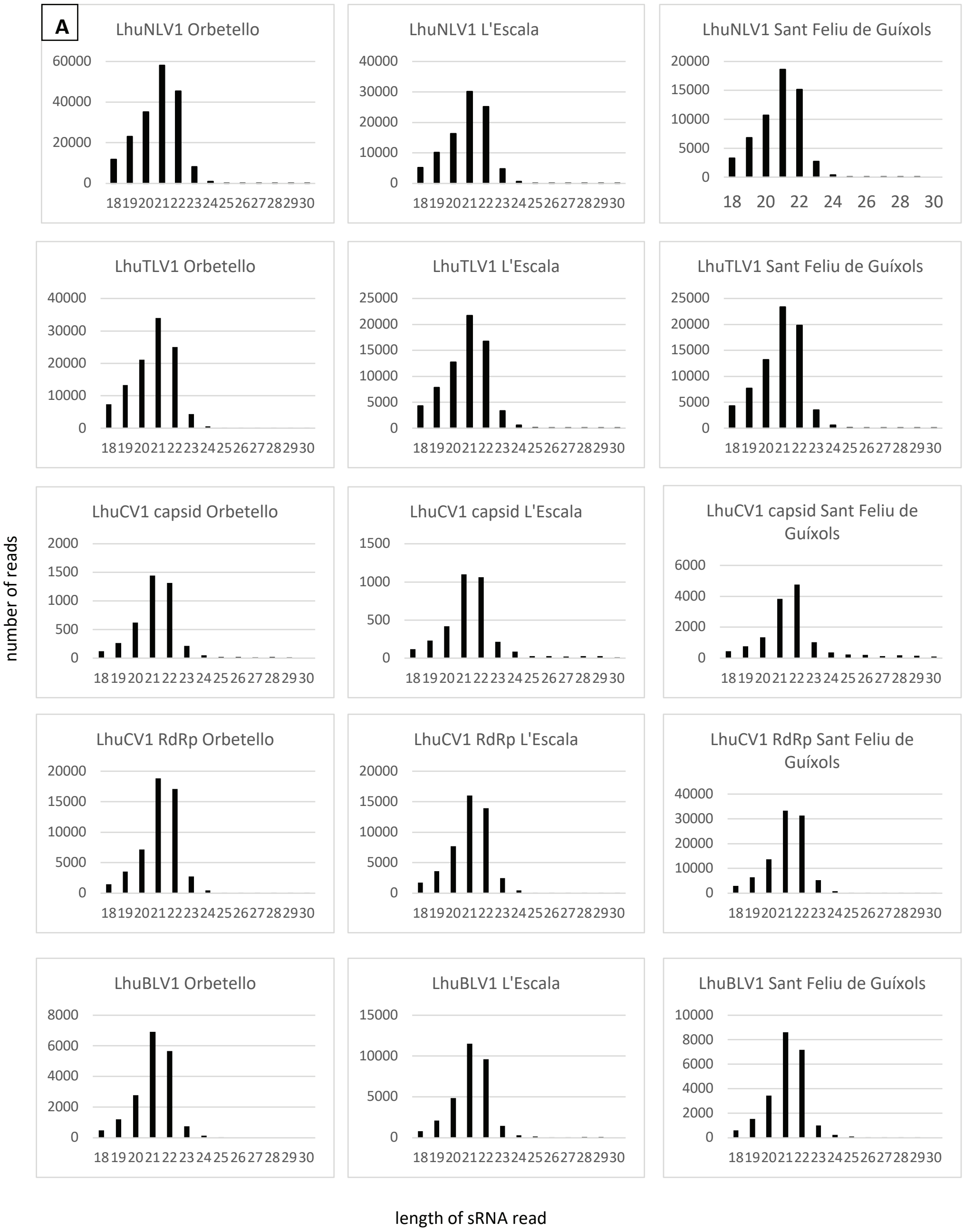

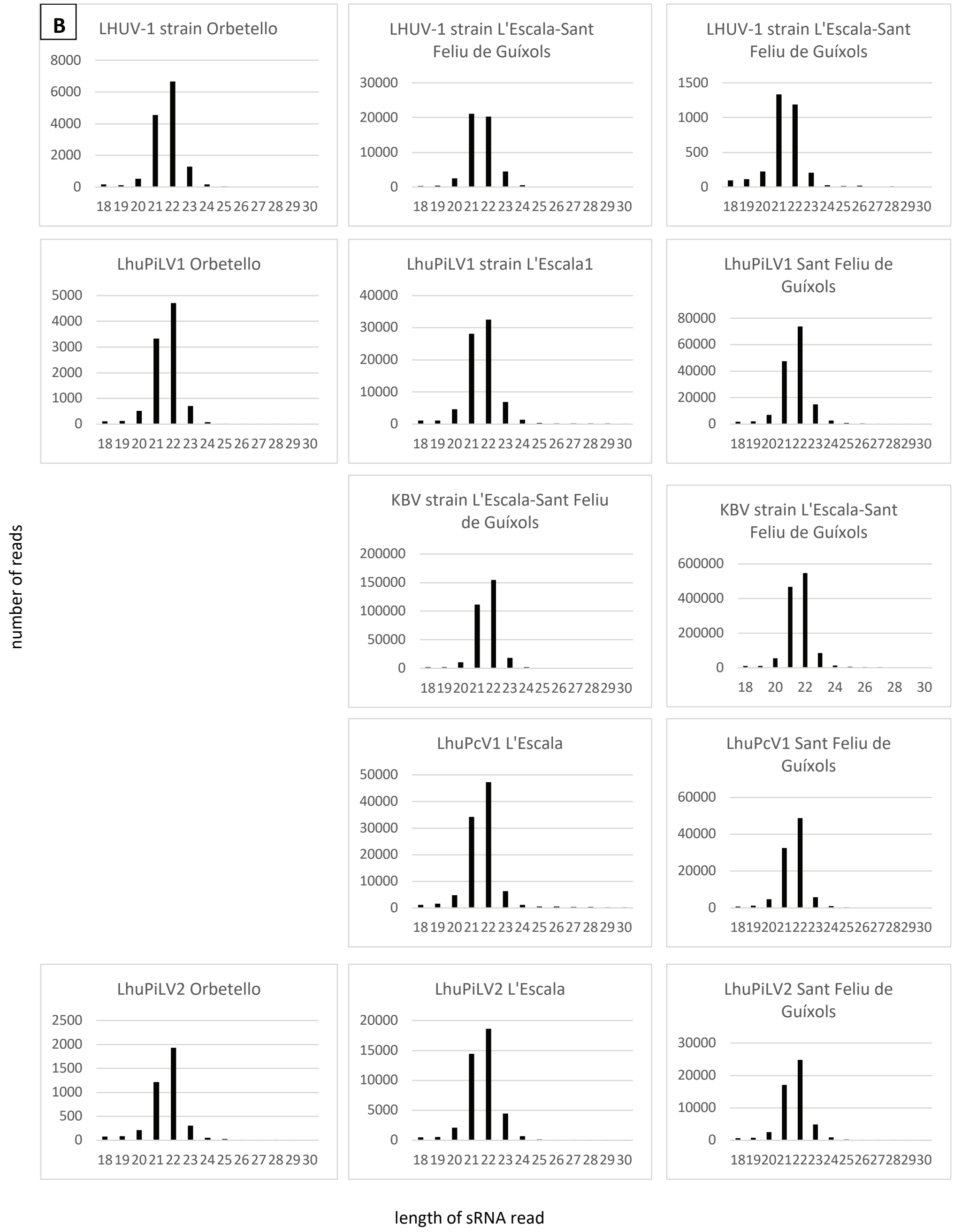

Supplementary Figure 3. Diversity of small RNA (sRNA) size distributions in *Li. humile.* Comparison of the sRNA size distribution of all viruses causing an RNAi response in at least two populations of *Li. humile* shows a peak mainly at 21 nt (A) or 22 nt (B) consistently across populations (populations 1: Orbetello, left; 2: L'Escala, middle; 3: Sant Feliu de Guíxols, right).

Supplementary Figure 4

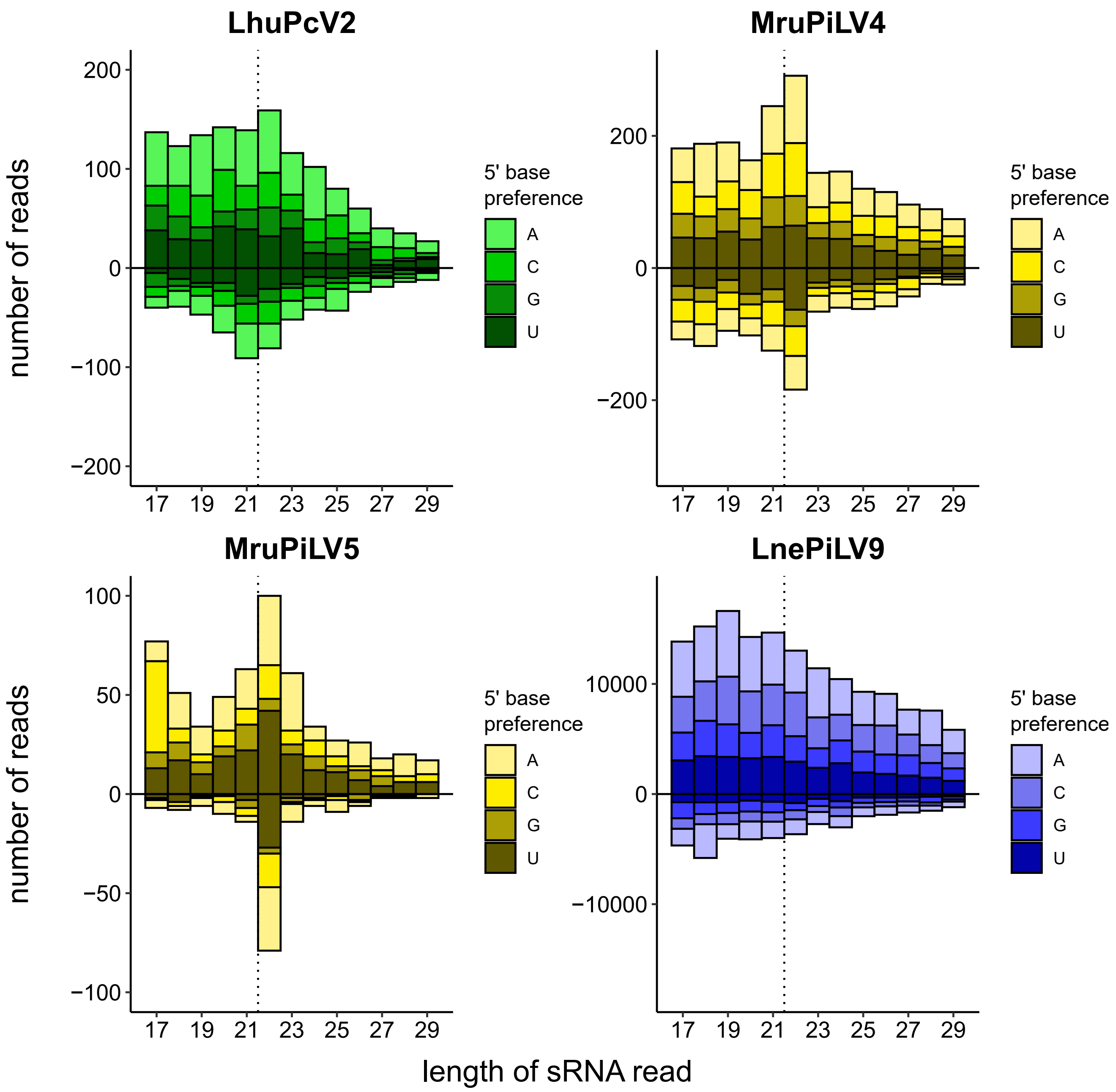

Supplementary Figure 4. Atypical sRNA distributions. One virus infecting *Li. humile* (green) and two infecting *M. rubra* (yellow), showed atypical, broader sRNA distribution around main peaks at 21-22 nt with an overall low sRNA read count. One virus infecting *La. neglectus* (blue) showed a broad sRNA size distribution in the absence of a main size class, at a high sRNA count; dotted vertical line separates 21 nt and 22 nt position. For each virus, the 5'-base preference is given, showing that Guanine (G) was significantly underrepresented in positive and negative strands (ChiSquare tests; all p-values < 0.001). In all four viruses, positive strands were overrepresented compared to negative strands.
